# Functions of vitamin B3 and its metabolites in epigenetic regulation

**DOI:** 10.1039/d6cb00165c

**Published:** 2026-10-09

**Authors:** Nolan D. Swain, Annie Gao, Y. George Zheng

**Affiliations:** a Department of Pharmaceutical and Biomedical Sciences, College of Pharmacy, University of Georgia Athens Georgia 30602 USA yzheng@uga.edu; b Department of Civil and Environmental Engineering, University of California Berkeley California 94720 USA

## Abstract

Recent research advances point toward an intimate connection between metabolic chemistry and molecular mechanisms of epigenetic gene regulation that modulates cellular functions. Many small molecule metabolites generated through metabolic pathways are demonstrated to have the ability or potential to bind or modify nuclear chromatin, chromatin-modifying enzymes, or other chromatin associated proteins. These processes cause alterations of chromatin dynamics, gene expression, and other DNA-templated functions, which ultimately lead to phenotypic changes of the cell. Vitamin B3 is an essential micronutrient of the human diet required for normal physiological function, but the dysregulation of vitamin B3 metabolism has been found in association with several pathological conditions. Intracellularly, vitamin B3 is metabolized into various intermediate metabolites, several of which are functional molecules that have been found to exert an influence on epigenetic regulation of gene function. Multiple lines of experimental evidence show that vitamin B3, and these functional metabolites, can directly or indirectly influence histone post-translational modification, DNA methylation, and alter chromatin remodeling and gene expression. The appreciation of vitamin B3's roles in epigenetic regulation provides a novel framework for understanding diet- and metabolism-driven mechanisms of gene program control. In this review, we discuss the current understanding of vitamin B3 metabolic pathways, biochemical characteristics of various B3-derived intermediates, and their distinctive mechanisms in epigenetic regulation concluding with a reflection on problems, challenges, and prospects in this field.

## Abstract 2

Vitamin B3 and its metabolites, generated by human and mammalian metabolism, exert considerable epigenetic influence, as reviewed here.

## Graphical abstract

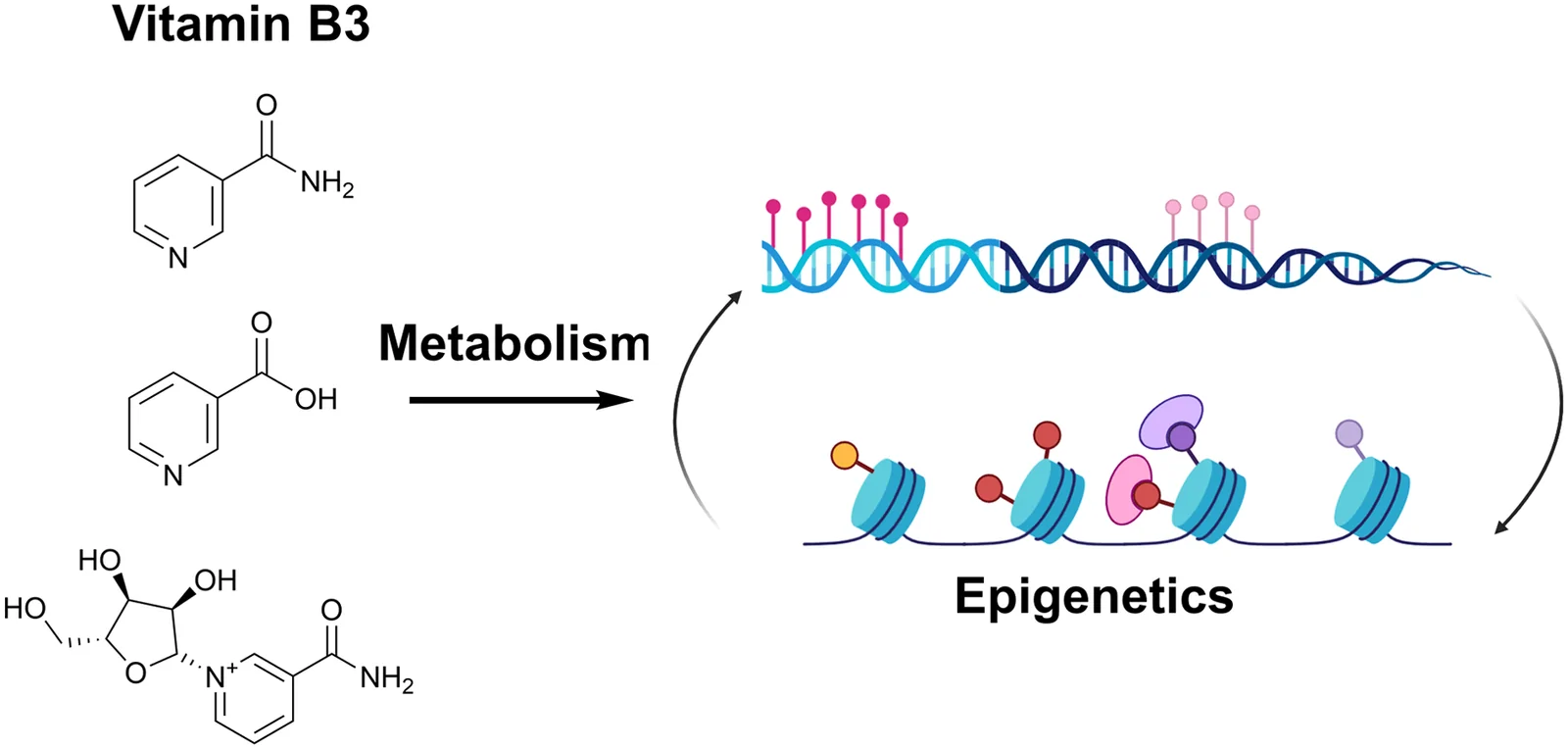

## 1. Introduction

Vitamin B3 collectively refers to three organic molecules, nicotinic acid, nicotinamide, and nicotinamide riboside (NR), each of which contains either a pyridine-3-carboxamide or pyridine-3-carboxylic acid functional group and serves as a precursor for the biosynthesis of nicotinamide adenine dinucleotide (NAD^+^) and nicotinamide adenine dinucleotide phosphate (NADP^+^) (Fig. 1).^1^ These three chemical species together are often referred to as niacin, which is an essential micronutrient required to maintain normal human physiology predominantly by the metabolic conversion of these molecules to NAD^+^ and NADP^+^. NAD^+^ and NADP^+^, along with their reduced forms NADH and NADPH, are essential cofactors required for the function of many cellular enzymes and play key roles in a wide range of biological functions such as glycolysis, cellular respiration, oxidative phosphorylation, the citric acid cycle, and protein post-translational modification (PTM) among others.^2–4^ Additionally, NAD^+^ and NADP^+^ serve as key regulators of several redox reactions within cells placing them as vital biological molecules to maintain cellular oxidative homeostasis.^5^ In addition to NAD^+^ and NADP^+^, vitamin B3 metabolism produces a considerable number of downstream metabolites with varying functional roles within cells.^6^

**Fig. 1:**
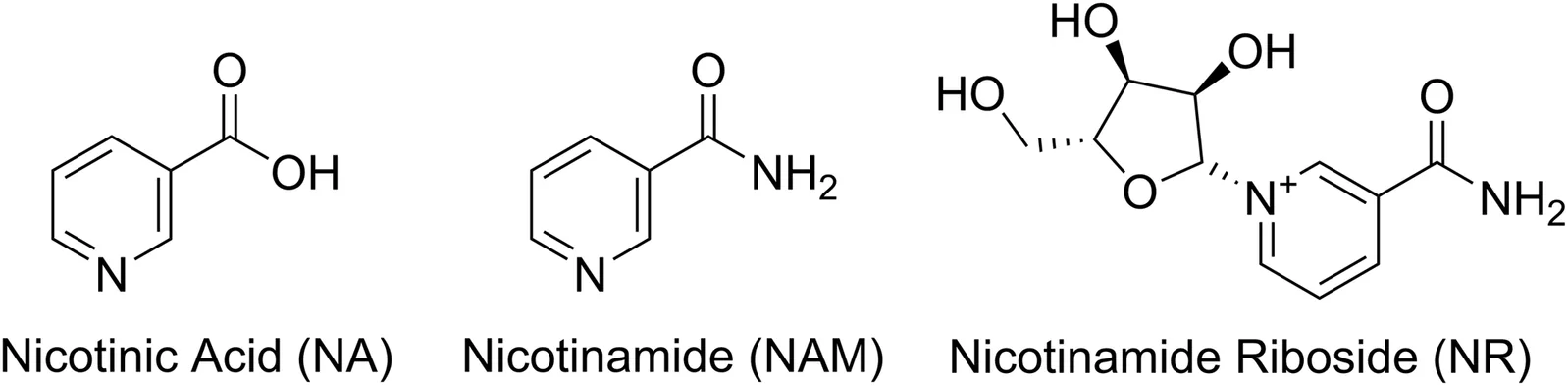
Chemical structures of vitamin B3.

Epigenetic mechanisms of gene regulation have long been understood as a crucial process by which cells can modulate RNA and protein levels *via* heritable structural alterations to chromatin and DNA without changes to the DNA sequence.^7^ The National Institutes of Health (NIH) considers epigenetics as “…the study of changes in the regulation of gene activity and expression that are not dependent on gene sequence,” and includes “…heritable changes in gene activity and expression (in the progeny of cells or of individuals) and also stable, long-term alterations in the transcriptional potential of a cell that are not necessarily heritable.”^8^ Both heritable and nonheritable changes to gene expression or transcriptional potential are primarily controlled by genomic DNA methylation, PTM of nuclear histone proteins that form the core structure of the nucleosome, and several non-coding RNA-mediated processes.^9^ Through these epigenetic mechanisms, genes can be selectively activated or silenced as required by cellular needs leading to short- or long-term alterations to RNA or protein levels and functional consequences for the cell.

Epigenetic regulation can therefore be interpreted as a set of cellular mechanisms that serve to dictate gene expression and transcription independent of the cell's DNA sequences generally through alteration of chromatin structure or gene accessibility. Considerable evidence demonstrates a clear intrinsic connection between cellular metabolism and epigenetic mechanisms of gene expression.^10–12^ Metabolites produced from cellular metabolism serve as cofactors and substrates for epigenetic modifying enzymes that attach or remove chemical groups on histones and other proteins along with DNA, while some metabolites may directly modify histones and DNA non-enzymatically.^10,13,14^ An understanding of the cellular metabolites and their influence on epigenetic programming of gene expression is crucial to building a holistic understanding of metabolism-driven mechanisms of gene regulation.

The metabolism of vitamin B3 generates numerous metabolites that have been identified to participate in epigenetic mechanisms; however, a comprehensive understanding of this phenomenon is lacking. Here, a critical examination of vitamin B3 biochemistry and mammalian vitamin B3 metabolism is provided, along with an in-depth review of the individual metabolites produced from vitamin B3 metabolism and the epigenetic regulatory effects these compounds elicit.

## 2. Vitamin B3 and human health

Vitamin B3, as an essential micronutrient, is an indispensable component of the human diet and is required to sustain normal homeostatic function as evidenced by pellagra, a debilitating condition caused by vitamin B3 deficiency that is fatal if left unremedied.^1^ The study of pellagra's etiology led to the discovery of nicotinic acid in 1937, the first form of vitamin B3 identified that was designated as a pellagra-preventing factor.^15^ Ultimately, pellagra's pathology arises from a lack of intracellular NAD^+^ caused by insufficient dietary niacin intake or the inability to generate NAD^+^ from vitamin B3.^16^ Clinical manifestation of pellagra is characterized typically by the “four D's,” including dermatitis, diarrhea, dementia, and eventually death if untreated.^17^ Without adequate nutrition to fulfil dietary requirements necessary for NAD^+^ biosynthesis, several essential pathways like cellular respiration, glycolysis, protein synthesis, and others begin to breakdown leading to the widespread adverse effects that present with pellagra. Generally, tissues with higher respiration rates are more susceptible to the effects of pellagra, highlighting why the central nervous system is so greatly affected and dementia presents.^18^ This fatal deficiency underscores the importance of vitamin B3 in human health and considerable research efforts to document its metabolism and role in human biology and cellular physiology since the early 20th century has provided an in-depth understanding of the enzymes and intermediates generated from vitamin B3 metabolism.

## 3. Biochemistry of vitamin B3 metabolic pathways

### 3.1. Pathways of NAD^+^/NADP^+^ biosynthesis from vitamin B3

The three chemically distinct forms of vitamin B3 can all be accessed by mammalian cells for the biosynthesis of NAD^+^ and NADP^+^; however, due to the different chemical structures of each form several convergent pathways exist to coordinate their conversion (Fig. 1). Upon ingestion, absorption of vitamin B3 predominantly occurs in the stomach and small intestine where several transporters exist for uptake.^19^ In the intestinal tract, sodium-coupled monocarboxylate transporter 1 (SMCT1, SLC5A8) and SMCT2 (SLC5A12) primarily are responsible for uptake.^20,21^ Hepatic and renal absorption of vitamin B3 can also occur *via* organic anion transporter 2 (OAT2, SLC22A7) and OAT10 (SLC22A13) respectively. At low concentrations, carrier-mediated facilitated diffusion is the predominant mechanism of transport; however, saturating high-doses likely induce passive diffusion across the cell membrane.^19,22^ NAD^+^ production from nicotinic acid utilizes the Preiss–Handler pathway whereby imported nicotinic acid is converted to nicotinic acid mononucleotide (NAMN) by the action of nicotinic acid phosphoribosyltransferase (NAPRT) (Fig. 2).^23,24^ NAMN is then converted by nicotinamide mononucleotide adenylyltransferase (NMNAT) to nicotinic acid adenine dinucleotide (NAAD).^25^ The nicotinic acid of NAAD is then amidated by NAD synthetase (NADS) to complete the conversion to NAD^+^.^26^

**Fig. 2:**
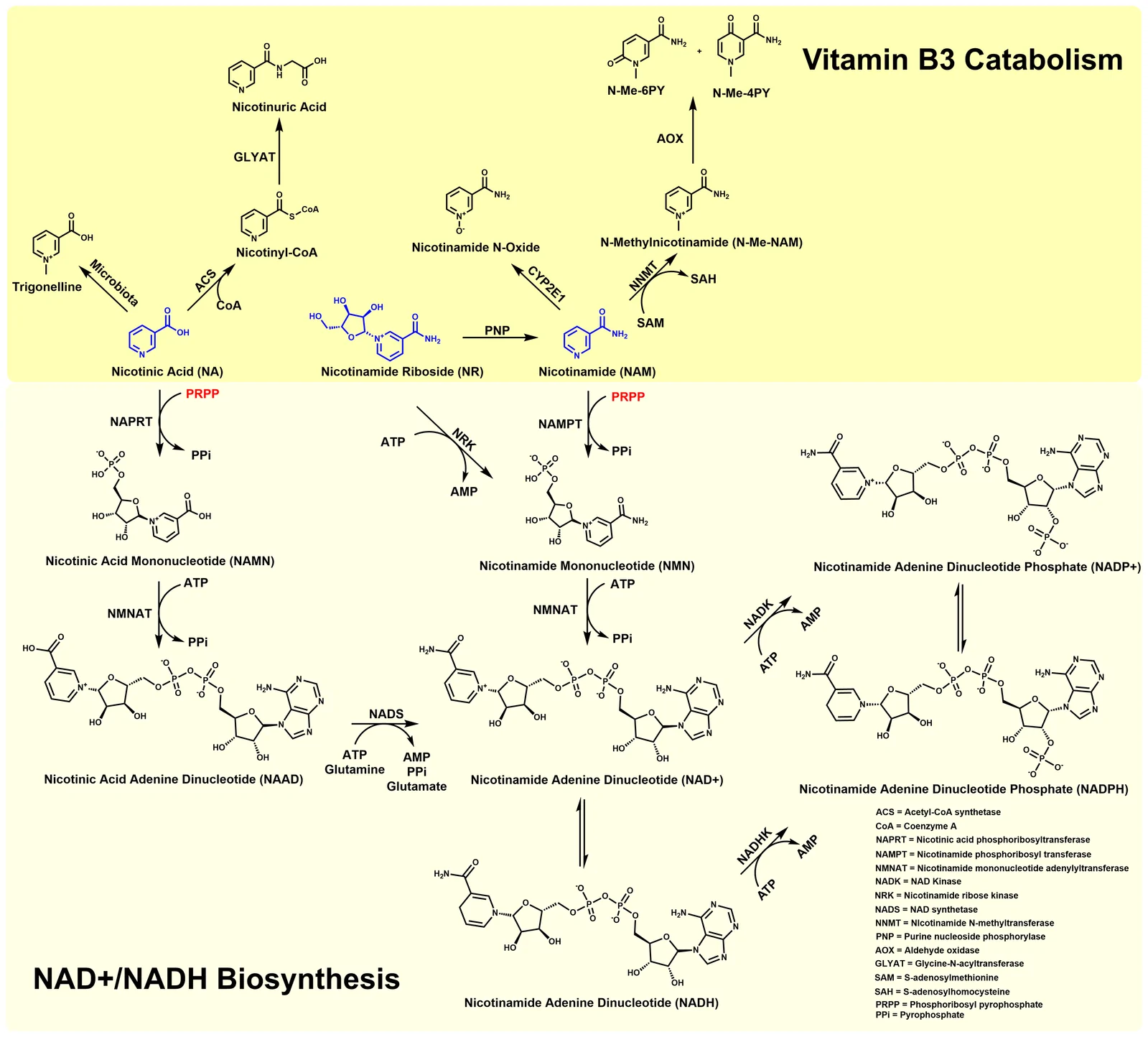
Metabolic pathways of vitamin B3. Top section (dark yellow) depicts catabolism. Bottom section (pale yellow) depicts biosynthetic pathways for NAD^+^/NADH and NADP^+^/NADPH.

The nicotinamide form of vitamin B3 follows the salvage pathway, a two-step process with the first step being the conversion to nicotinamide mononucleotide (NMN) by the action of nicotinamide phosphoribosyltransferase (NAMPT).^27^ In the second step, NMN is converted to NAD^+^ by the ATP-consuming enzyme nicotinamide mononucleotide adenylyltransferase (NMNAT).^25^ This salvage pathway is the primary means by which cells maintain the intracellular pool of NAD^+^ and as referenced by the name serves as a mechanism for cells to salvage nicotinamide generated by NAD^+^-consuming enzymes and other cellular processes back into NAD^+^. The nicotinamide riboside form of vitamin B3 undergoes phosphorylation by nicotinamide riboside kinase (NRK) to form NMN which is then converted by NMNAT to NAD^+^ like in the nicotinamide salvage pathway.^28^ Nicotinamide riboside can separately be converted by the action of purine nucleoside phosphorylase (PNP) enzymes into nicotinamide.^29,30^

NADP^+^ is generated from NAD^+^ by the enzyme NAD kinase (NADK) that phosphorylates NAD^+^.^31^ NADPH can be produced by the action of NADH kinase (NADHK) to phosphorylate NADH; however, the bulk of intracellular NADPH is generated from the reduction of NADP^+^ by several metabolic dehydrogenase enzymes.^32^ An important distinction between the biosynthesis of NAD^+^ using nicotinic acid and nicotinamide *versus* that of nicotinamide riboside is the pathways’ reliance on phosphoribosyl pyrophosphate (PRPP). The NAPRT and NAMPT enzymes require PRPP as a cofactor for the reaction making nicotinic acid and nicotinamide PRPP-dependent precursors to NAD^+^ whereas NRK does not rely on PRPP making nicotinamide riboside PRPP-independent. Respectively, NAMPT, NAPRT, and NRK catalyze the committed steps of NAD^+^ biosynthesis from nicotinamide, nicotinic acid, and nicotinamide riboside.

In concert, all these pathways regulate the pool of NAD^+^/NADP^+^ and their reduced forms, along with the various catabolites depending on the organ, tissue, and cell type. Depending on the cell-type, the context-dependent needs, and the whole-body dietary status, the flux through these pathways can vary dramatically. Tissue turnover rate of NAD^+^ is a stark example, where turnover in the small-intestine is ∼0.2 hours, a 10-fold faster turnover rate than any other tissue.^33^ Tissue-specific demands and enzyme expression levels likely dictate flux through the vitamin B3 metabolic pathways. Additionally, sub-cellular compartmentalization of these metabolic pathways occurs with pathways existing in the cytosol, mitochondria, and nucleus to differentially coordinate conversion of metabolites as needed.^34^

### 3.2. Catabolism of vitamin B3

Vitamin B3 catabolism produces a considerable variety of metabolites depending on the vitamer. Beginning with nicotinic acid, excess amounts are conjugated to glycine *via* the glycine-conjugation pathway to produce nicotinuric acid in the liver, the primary site of nicotinic acid metabolism and NAD^+^ biosynthesis.^35^ First, in this process nicotinic acid is covalently attached to coenzyme A (CoA) by acetyl-CoA synthetase (ACS) to produce the reactive nicotinyl-CoA species. Nicotinyl-CoA is then reacted upon by glycine-*N*-acyltransferase (GLYAT) to generate nicotinuric acid for excretion. Nicotinic acid uptake into liver hepatocytes in HepG2 and human primary hepatocytes seems to rely on a specific, high-affinity, and pH-dependent carrier-mediated system.^36^ Mathialagan *et al.* (2020) found that nicotinic acid transport into cells relied on organic anion transporter (OAT) 10 (SLC22A13) suggesting the bulk of dietary-obtained nicotinic acid is absorbed through this transporter.^36,37^ The liver is rich in ACS enzymes providing the optimal environment for metabolism of nicotinic acid through this pathway.^38^ Another potential route of nicotinic acid metabolism generates *N*-methyl-nicotinic acid also called trigonelline. This catabolite has been observed in humans; however, the exact mechanism that generates this compound is uncertain.^39,40^ A potential mechanism is the metabolization of nicotinic acid by the gut microbiota as demonstrated in a metabolomic study of gut flora-derived metabolites in obese humans.^41^

Under most circumstances, nicotinamide generated by NAD^+^ degradation and NAD^+^ consuming enzymes is recycled back into NAD^+^*via* the salvage pathway. However, when excess nicotinamide is present, two separate clearance pathways can be utilized. The first uses the enzyme CYP2E1, also located in the liver, to oxidize nicotinamide to nicotinamide *N*-oxide which is then excreted in urine and can be found in circulation.^42^ This pathway occurs less commonly and CYP2E1 is used for metabolism of many other endogenous substrates. The other, more commonly accessed clearance pathway, uses the enzyme nicotinamide *N*-methyltransferase (NNMT) to methylate nicotinamide at the pyridine nitrogen forming *N*-methyl-nicotinamide (N-Me-NAM).^43^ This reaction utilizes the important biomolecule *S*-adenosylmethionine (SAM) as a co-substrate that donates methyl groups forming *s*-adenosyl homocysteine (SAH) and N-Me-NAM. Thus, high niacin intake may lead to a depletion of labile methyl groups and an elevation of plasma homocysteine (Hcy), a phenomenon termed hyperhomocysteinemia, which is often seen in patients with cardiovascular disease (CVD).^44^

From N-Me-NAM, several oxidized products can be generated by aldehyde oxidase (AOX) enzymes based on the position of the introduced carbonyl relative to the carboxamide.^45^ The predominant metabolites produced from this reaction are *N*-methyl-3-carboxamide-6-pyridone (N-Me-6PY) and *N*-methyl-3-carboxamide-4-pyridone (N-Me-4PY). Within the literature some ambiguity exists regarding the nomenclature of these compounds with N-Me-6PY sometimes referred to as N-Me-2Py, but for the sake of this review we will use N-Me-6PY. Under normal physiological conditions N-Me-6PY is the main product of NAD^+^ degradation and has been identified as a uremic toxin that inhibits poly(ADP-ribose) polymerase (PARP) enzyme activity.^46^

## 4. Effects of vitamin B3 metabolites on epigenetics

Given the central role of vitamin B3 metabolism in cellular physiology and NAD^+^ biosynthesis, it is unsurprising that this vitamin and its metabolites exert considerable influence over numerous epigenetic mechanisms of gene regulation. Beyond NAD^+^, many of the metabolites from vitamin B3 have been identified as regulators of epigenetic functions through their action on several different epigenetic modifying enzymes and pathways that will be reviewed here. First, an initial overview of basic epigenetic mechanisms is provided, after which more detailed specific accounts of the epigenetic effects of select metabolites from the vitamin B3 metabolome are discussed.

### 4.1. Epigenetics and chromatin modifications

Of particular importance to our understanding of epigenetic mechanisms is the post-translational modification (PTM) of histone proteins located within the nucleus of cells. Histone proteins constitute the core of the nucleosome, forming the structural scaffold that organizes chromatin and thereby governs genome architecture and accessibility. Due to their integral role in chromatin and genome organization, histones are foundational to gene expression with their chemical and physical properties exerting profound effects on regulation of gene expression. Generally, the nucleosome structure consists of two copies of 4 histone proteins each, H2A, H2B, H3, and H4, that are joined to form an octamer wrapped with a 147 bp segment of DNA.^47^ These histone proteins contain long, flexible, N-terminal tails abundant in lysine and arginine residues that are the site of extensive PTM by both enzyme-catalyzed and non-enzymatic mechanisms that covalently attach a diverse set of chemical moieties at these residues.

Most commonly, histone PTM is regulated by “writer” enzymes that catalyze the covalent addition to proteins while the removal of these PTMs is regulated by “eraser” enzymes.^48^ Effectively, these PTMs can dramatically shift the chromatin landscape and subsequently gene expression by directly altering the structure of chromatin *via* alteration of the charge on histones or by serving as markers for the recruitment of “reader” proteins that recognize these PTMs to remodel chromatin or carry out effector functions at the sites of modification.^49–51^ For example, acetylated lysine sites on histones lose their positive charge thereby weakening the interaction between the negatively charged DNA and the histone protein causing a relative loosening in the chromatin structure and an increase in chromatin accessibility. Whether by altering chromatin structure or serving as a recruitment marker, histone PTMs function as gene repression or gene activation marks based on the specific PTM and site of modification. Importantly, the enzymes responsible for regulating histone PTM addition and removal respond dynamically to changes in metabolism as many of these enzymes’ functions rely on metabolic intermediates as cofactors or cosubstrates.^10^ Consequently, the vitamin B3 metabolome and alterations to it will affect this PTM-mediated mechanism of epigenetic gene regulation.

DNA methylation is widely recognized as one of the predominant epigenetic mechanisms responsible for the regulation of genes with a longer history of study than histone PTM.^52,53^ The process of DNA methylation involves the methylation of cytosine to 5-methylcytosine (5mC) by the action of a family of DNA methyltransferase enzymes (DNMTs) using SAM as a methyl-donor.^54^ Like histone PTM, DNA methylation is a dynamic process regulated by the coordinated action of writer, eraser, and reader enzymes to coordinate gene expression. 5mC is highly enriched within the human genome, but its function is context-dependent and has been associated with both gene repression and activation.^55,56^ Generally, DNA methylation marks reduce the ability for transcription factors to bind at sites of methylation thereby decreasing the expression of genes controlled by sites of DNA methylation. 5mC marks within the genome can be maintained through DNA replication and some studies implicate the potential for transgenerational inheritance although this process is still highly debated regarding its prevalence and impact in mammals.^57–59^

### 4.2. Epigenetic influence through nicotinamide (NAM)

Nicotinamide is the amide form of vitamin B3 and the predominant circulating form in humans obtained from the diet and generated by NAD^+^-consuming enzymes like poly(ADP-ribose) polymerases (PARPs), sirtuins (SIRTs), and NAD^+^ glycohydrolases. Abundant in many foods like fish, meat, legumes, and grains or cereals, nicotinamide is a common compound found in the human diet.^1^ Recommended daily intake of nicotinamide is ∼20 mg per day or ∼0.3 mg per kg per day and is a highly tolerated compound with adverse effects not observed until ∼3000 mg per day with most individuals consuming more than the daily recommendation.^60,61^ As a precursor to NAD^+^, nicotinamide has been demonstrated to promote DNA repair responses, cellular energy production, and contribute to clearance of reactive oxygen species.^62^

The nicotinamide form of vitamin B3 directly exerts influence over epigenetic pathways of gene regulation by participating in several enzymatic reactions as either an inhibitor or a substrate of the reaction. Of particular importance, is the ability of nicotinamide to serve as a pan-inhibitor of the class III histone deacetylase enzymes also referred to as sirtuins (SIRTs). SIRTs are a family of seven NAD^+^-dependent enzymes (SIRT1–SIRT7) that utilize a single molecule of NAD^+^ to catalyze the removal of acetyl and other short-chain acyl groups from lysine residues generating 2′-*O*-acyl-ADPR and nicotinamide.^63^ SIRTs play a key role in epigenetic regulation through their eraser enzyme function with the ability to remove acyl groups from both histone and non-histone proteins giving them significant control over chromatin dynamics and gene transcription.^64^

The activities of SIRTs are strongly linked to cellular metabolic processes through their dependence on NAD^+^; however, excess nicotinamide within the system functions to inhibit the SIRT reaction. This relationship intrinsically links vitamin B3 and its metabolism to SIRT enzymatic activity and subsequently mechanisms of gene regulation through a feedback mechanism.^65^ Ample evidence has defined the role of SIRTs as critical epigenetic modifying enzymes and nicotinamide's IC_50_ towards the members of the SIRT family range from 20 µM to 400 µM indicating a highly varied inhibition effect.^66,67^ With such diverse IC_50_ values, fluctuations in intracellular nicotinamide concentrations are likely to have a considerable influence on the enzymatic activity of different SIRT members.

Like the SIRT-catalyzed reaction, the PARP-catalyzed reaction consumes NAD^+^ and produces nicotinamide that also functions as a product inhibitor. Nicotinamide is a fairly potent inhibitor of the PARP-catalyzed reaction, measured to have inhibiting effects at sub-millimolar concentrations.^68–70^ PARPs are an enzyme family consisting of 17 proteins in total with four subfamilies that catalyze the transfer of ADP-ribose units from a donor NAD^+^ to targeted substrates.^71^ This general process is known as ADP-ribosylation, a type of PTM that includes both poly-ADP ribosylation (PARylation), where multiple ADP-ribose units form long linear or branched chains of poly-ADP-ribose, and mono-ADP ribosylation (MARylation) where single ADP-ribose units are covalently attached primarily to protein substrates; however DNA and small molecule compounds have been identified as substrates as well.^72,73^ Both PARylation and MARylation are widespread PTMs within cells generally implicated in DNA-damage responses, maintenance of chromatin structure, chromatin dynamics, and as a recruitment marker for protein docking.^74–77^ PARP enzymes and the epigenetic effects of PARP inhibition are discussed elsewhere in this review (see Section 4.8), so no additional discussion is added here. The later discussed effects of PARP1 inhibition by other biomolecules are likely shared with nicotinamide, but this requires experimental validation to confirm.

Nicotinamide has another interesting function within cellular metabolism that has only recently been appreciated for its ability to regulate epigenetic mechanisms of gene expression. As previously discussed, excess nicotinamide is cleared beginning with its enzymatic methylation by NNMT to generate N-Me-NAM. This consumes a molecule of SAM and produces a molecule of SAH in the process. Importantly, SAM functions as a co-substrate for many critical methylating epigenetic enzymes like protein arginine methyltransferases (PRMTs), histone lysine methyltransferases (KMTs), and DNMTs where it serves as the methyl-donor for these enzyme-catalyzed reactions that coordinate a diverse set of epigenetic responses.^78^ A considerable body of evidence has accumulated to suggest that the methylation of nicotinamide by NNMT to form N-Me-NAM, serves as a “methyl sink” that consumes a significant proportion of intracellular SAM.^79–81^ This methyl-sink effect dramatically alters the epigenetic landscape of a cell by shifting the SAM/SAH ratio and the intracellular concentration of nicotinamide. This affects epigenetic mechanisms in two ways. First, it reduces SAM availability for use as a substrate in enzyme-catalyzed reactions. Second, it raises SAH levels. Because SAH acts as a competitive inhibitor at the SAM binding site of methyltransferase enzymes, SAH accumulation will directly inhibit these enzymes. Likely, depletion of methyl-donors by NNMT prevents the transfer of methyl groups to targeted substrates by PRMT-, KMT- and DNMT-catalyzed methylation reactions limiting the activity and epigenetic modulating effect of these enzymes.

First reports of NNMT's methylation sink potential were discovered in aggressive cancer cell lines with a connection observed between the expression levels of NNMT and the relative aggressiveness of the cancer.^79^ Enhanced expression of NNMT is associated with reduced methylation potential in ovarian cancer cells, a decrease in several histone methylation marks like H3K9me2 and H3K27me3, and a corresponding alteration to the expression of several cancer-related genes. The altered methylation sites are likely controlled by methyltransferases with higher *K*_m_ values for SAM that would be more sensitive to NNMT activity alterations. The ability for NNMT to regulate histone methylation has also been observed in studies with naïve and primed human embryonic stem cells (hESCs).^82^ Compared to the naïve state, primed hESCs have enhanced SAM levels, along with depleted N-Me-NAM levels. This curious observation is explained by the fact that naïve hESCs have ∼20–285 fold-more NNMT as measured by mRNA expression compared to the primed state. The increase in NNMT levels, and subsequent increased nicotinamide consumption, correlates with specific alterations to epigenetic marks. Immunoblotting shows significant changes to H3K27me3 and H3K9me3 levels without significant changes to the levels of KMTs or lysine demethylases that regulate these histone marks. The most likely explanation is that depletion of SAM and nicotinamide through the methyl sink effect driven by increased NNMT levels limits the catalytic activity of several methyltransferase enzymes that regulate these histone marks.

Beyond histone methylation, additional studies have demonstrated that alterations to NNMT enzyme activity modulate DNA methylation and epigenetic signaling mechanisms. Takahashi *et al.* (2022) documented this phenomenon by demonstrating in mouse model studies of chronic kidney disease (CKD) that nicotinamide levels are significantly depleted and that N-Me-NAM, N-Me-4PY, and N-Me-6PY levels increase compared to healthy mice. This observation suggests that some alterations to NNMT levels or activity in CKD promote depletion of nicotinamide and SAM as evidenced by the increased NNMT reaction products and decreased substrates.^83^

NNMT-KO mouse models show an increase in SAM and methylation at CpG islands associated with the CTGF gene compared to wild-type suggesting NNMT activity alterations do indeed influence DNA methylation levels. In support of this conclusion, several studies have demonstrated that nicotinamide supplementation induces changes to DNA methylation status. Li *et al.* (2013) demonstrated that rats supplemented with 1 g kg^−1^ and 4 g kg^−1^ of nicotinamide for 8 weeks had ∼1.5-fold and ∼6-fold increases in N-Me-NAM levels respectively and a significant decrease in global DNA methylation in rats receiving 4 g kg^−1^.^84^ Along with the global DNA hypomethylation, methylation at CpG sites in the promoter regions of Nnmt and Tp53 was specifically found to be decreased, indicating the potent epigenetic modulation effects of nicotinamide.

A comparable mechanism was observed *in vivo* with maternal administration of nicotinamide in rats. Significant decreases in placental and fetal liver levels of DNA methylation can be found in rats fed nicotinamide compared to the control.^85^ Both 1 g kg^−1^ and 4 g kg^−1^ supplementation with nicotinamide induced significant global DNA hypomethylation with total DNA cytosine methylation in the fetal liver decreasing ∼59%. Additionally, this DNA hypomethylation in the liver is correlated with increases to mRNA expression levels of Nnmt and Dnmt-1. The findings of Li *et al.* (2013) suggest that increased Nnmt expression levels may be due to hypomethylation at the promoter of this gene allowing for enhanced transcription. Increased mRNA expression levels of Dnmt-1 may be a compensatory mechanism attempting to rescue the DNA hypomethylation associated with nicotinamide supplementation. Taken together with the previous study, the evidence is clear that nicotinamide and NNMT profoundly influence DNA methylation and subsequently, epigenetic mechanisms of gene regulation and signaling.

Nicotinamide is a potent epigenetic regulating molecule through its ability to participate in NAD^+^ biosynthesis, its function as a product feedback pan-SIRT inhibitor, and as an inducer of intracellular methyl sinks by NNMT-catalyzed methylation (Table 1). It is interesting that by serving as a precursor to NAD^+^ biosynthesis, and therefore an activator of SIRT enzymes, nicotinamide supplementation can potentially promote SIRT activity despite functioning as an inhibitor of the reaction. Several studies have documented this observation that cellular administration of nicotinamide can paradoxically enhance SIRT activity despite serving as a direct inhibitor of this reaction.^86,87^ This phenomenon has been reviewed elsewhere in extensive detail and is a significant effect that should not be overlooked.^88^ Nonetheless, this is an important consideration that must be kept in mind when studying nicotinamide's effects on epigenetics and further complicates the efforts to deconvolute these pathways and the precise mechanistic outcomes.

**Table 1:** Summary of global and site-specific effects of nicotinamide and nicotinamide riboside through the NNMT-induced methyl-sink effect

Methyl-sink global *vs.* site-specific effects		
	Global	Site-specific
Nicotinamide and NNMT	• Global asymmetric dimethylarginine decrease^79^	• H3K9me1, H3K9me2, and H3K27me3 decrease^79^
	• Global DNA methylation decrease^84,85^	• H3K4me3 and H3k27me3 decrease^81^
	• PP2a methylation decrease^79^	• H3K9me3 and H3K27me3 decrease^82^
		• CTGF CpG site methylation decrease^82^
Nicotinamide riboside and NNMT	• Global DNA methylation decrease^90^	• PGC-1α, NMNAT3, NRF1, and PPARγ CpG site methylation decrease^90^
		• Slc27a1 and Prdm16 CpG site methylation decrease^91^

### 4.3. Epigenetic influence through nicotinamide riboside

Nicotinamide riboside (NR), a member of the vitamin B3 family discovered to exist in mammals in 2003, and the only of the three with a sugar moiety, has several potential epigenetic modulating roles that have been identified.^28^ Likely, due to the sugar present, NR is uniquely taken up into cells by the equilibrative nucleoside transport (ENT) family, specifically ENT1 (SLC29A1), ENT2 (SLC29A2), and ENT4 (SLC29A4).^89^ However, similar to nicotinamide, NR can deplete the intracellular concentration of the methyl-donor SAM promoting a methyl sink and reducing the overall methylation potential of the cell thereby disrupting methylation-specific epigenetic mechanisms. This likely occurs because intracellular NR can be converted by the action of purine nucleoside phosphorylase (PNP) enzymes into nicotinamide. Nicotinamide produced by PNP can be methylated by NNMT enzymes consuming SAM in the process as previously discussed. Additionally, it is possible that some, or all, the discussed epigenetic effects observed with nicotinamide supplementation in the previous section may be shared with NR supplementation due to the existence of an endogenous pathway that allows for the conversion of NR to nicotinamide; however, this requires experimental validation.

The ability for NR to alter methylation-specific epigenetic mechanisms through the methyl sink effect was originally identified in a study that compared monozygotic twins receiving long-term NR supplementation or a placebo.^90^ NR supplementation significantly increases SAH levels implying a reciprocal SAM depletion. Along with this, a tissue-specific reduction in global DNA methylation can be observed in muscle. This indicates potential decreases in DNMT activity due to SAM depletion. Interestingly, NR supplementation induces hypo- and hypermethylation at different sites; however, ∼60% of CpG sites are hypomethylated indicating an overall global hypomethylating effect. Significant hypomethylation at CpG islands is in PGC-1α, NMNAT3, and NRF1 genes with a corresponding increase in PGC-1α expression. On the other hand, hypomethylation of PAX7 and NAPRT genes is associated with repression of these genes demonstrating the difficulty of predicting the functional outcomes of DNA methylation on gene activation or repression. Ultimately, NR supplementation regulates DNA methylation at several genomic loci and alters gene expression, indicating a potent epigenetic modulating effect from the induced depletion of SAM by NR. Further experimental investigation is required to elucidate the methyltransferases that regulate these methylation sites, and how specifically NR and NNMT regulate the action of these methyltransferases.

Altered DNA methylation patterns, and the simultaneous changes in gene expression observed through *in vivo* human study, highlight the ability for NR to modulate epigenetic mechanisms with functional outcomes. A similar phenomenon was documented in neonatal mice supplemented daily with NR from postnatal day 2 to day 20.^91^ Mice were euthanized on day 164 to understand how NR supplementation during development impacts late life. The DNA methylation status of several measured CpG sites proximal to the Slc27a1 and Prdm16 genes is hypomethylated in response to neonatal NR supplementation. Along with this, an inverse relationship can be observed between DNA methylation and mRNA expression of Prdm16 with NR supplementation suggesting that NR promotes DNA hypomethylation and inhibition of Prdm16 expression. NR supplementation promotes downregulation of Dnmt3b, a DNA methyltransferase enzyme. Additionally, in further experiments with 3T3-L1 adipocytes the same repression of Dnmt3b can be observed along with Dnmt3l repression during NR supplementation. Furthermore, Tet3, the gene encoding an enzyme that regulates DNA demethylation, is induced by NR. In conditions of high NR, reduced DNA methyltransferase expression, coupled with enhanced Tet3 expression, plausibly compounds the hypomethylated state arising from SAM depletion, leading to a further enhanced state of chromatin hypomethylation.

Diverging from DNA methylation, NR also exhibits the ability to activate several SIRT enzymes critical to regulating epigenetic acetylation events and subsequently gene expression. As a precursor to NAD^+^, it is believed that NR supplementation activates NAD^+^-dependent enzymes like SIRTs, a hypothesis that has been confirmed *in vitro* and *in vivo*.^92^ NR supplementation promotes FOXO1 deacetylation in a SIRT1-dependent manner which promotes expression of FOXO1 target genes. Additionally, targets of SIRT3, Ndufa9 and SOD2 experience increased deacetylation in a SIRT3-dependent manner with NR supplementation. Surprisingly, despite the ability to activate SIRT1 and SIRT3, no activation of SIRT2 is observed with NR supplementation suggesting the specificity of this activation is not entirely understood. Studies to this point have not examined histone substrates in response to NR supplementation; however, it is expected that NR-dependent activation of SIRT1 and SIRT3 will promote the deacetylation of histone substrates and induce epigenetic changes as evidenced by the decreased acetylation of FOXO1 demonstrating the potential for NR-induced attenuation of protein acetylation.

To further the notion that NR can promote epigenetic changes through enhanced deacetylation, NR functions as an allosteric activator of SIRT5-catalyzed deacetylation on histone substrates.^93^ In biochemical assays, NR at a concentration of 1 mM improves the deacetylation activity of SIRT5 towards several synthetic peptide substrates, including H3K9ac, as much as 4-fold. The observed increase in deacetylation activity, however, does not correlate with an increase in the desuccinylation activity of SIRT5 that this enzyme also exhibits. The activating effect is both concentration-dependent and saturable, pointing to the existence of a distinct binding site on SIRT5 for NR. Additionally, the activation effect is unique to SIRT5 as NR is unable to activate SIRT1, SIRT2, SIRT3, and SIRT6 in a biochemical assay indicating a clear specificity.

Direct binding of NR to SIRT5, measured *via* isothermal titration calorimetry analysis, is dependent on the presence of NAD^+^. NR's activating effect is conserved in the closely related *E. coli* SIRT equivalent, CobB, where a 3-fold activation was measured in the presence of NR. Sequence alignment reveals that conserved residues Y102, Y105, and W222 are the unique residues that contribute to the ability of NR to uniquely activate SIRT5. NR-meditated SIRT5 activation, along with increased deacetylation, was observed on synthetic histone peptides and lysate extracted from HeLa cells; however, this effect has not been tested *in cellulo* or *in vivo*. To fully elucidate the relevance of this effect and the relationship between NR and epigenetic mechanisms of gene regulation, further targeted cellular or animal studies are necessary.

NR exerts considerable influence over several epigenetic mechanisms through the activation and stabilization of several SIRT enzymes, downregulation of Dnmt3b and Dnmt3l genes, and induction of a methyl sink effect that limits the activity of DNMT enzymes (Table 1). The possibility remains that some epigenetic effects reported with nicotinamide are shared with NR, as the existence of a metabolic pathway to convert NR to nicotinamide allows for flux through nicotinamide pathways beginning with NR. Future studies should seek to precisely examine the potential overlap or differences between epigenetic effects induced by nicotinamide and NR. Additionally, further experiments to elucidate the precise mechanisms through which NR can enhance SIRT1 and SIRT3 activity *in vitro* and *in vivo* are needed. The observation that in a biochemical assay NR cannot activate SIRT1 or SIRT3 implies a different mechanism than direct binding. Likely, NR is converted intracellularly to NAD^+^, promoting SIRT activity through its increased availability, but this requires experimental validation.

### 4.4. Epigenetic influence through nicotinic acid (NA)

The involvement of nicotinic acid in epigenetic regulation is still only partially defined, yet accumulating evidence points to specific epigenetic mechanisms through which it may regulate gene expression. Like nicotinamide and NR, the main function of nicotinic acid is for the biosynthesis of NAD^+^, suggesting supplementation with this compound can induce similar downstream effects as NR and nicotinamide with respect to NAD^+^-dependent epigenetic mechanisms. Like other NAD^+^ precursors, nicotinic acid has been shown to enhance SIRT enzyme activity *via* several mechanisms.

Firstly, as an NAD^+^ precursor, nicotinic acid supplementation promotes the formation of intracellular NAD^+^, the co-substrate needed for the SIRT enzymatic reaction thereby promoting SIRT-catalyzed reactions. This can be observed in peripheral blood mononuclear cells (PBMCs) exposed to irradiation, where supplementation with 15 µM nicotinic acid raises intracellular NAD^+^ levels by ∼1.4-fold. Acetylation of p53 is significantly reduced in nicotinic acid supplemented cells implicating activation of SIRT1.^94^ Nicotinic acid supplementation likely restores and maintains NAD^+^ levels and activates SIRT1 deacetylation during significant DNA damage events such as in the case of radiation exposure.

Levels of histone acetylation marks under the control of SIRT1 were not examined in this study and other downstream epigenetic effects stemming from NAD^+^ upregulation by nicotinic acid supplementation were not examined. However, the authors confidently embrace in their own words the possibility of chromatin restructuring by SIRT1 directly targeting histones. Evidence of enhanced protein deacetylation on p53 lends itself to the conclusion that histone deacetylation is also regulated by this mechanism as p53 acetylation correlates with histone acetylation when SIRT1 levels are modulated.^95^ While enhanced p53 deacetylation demonstrates SIRT1 enzyme activation, and p53 deacetylation correlates with histone deacetylation, determining whether and how histones are affected can only occur through direct measurement.

In a potentially compounding mechanism, nicotinic acid treatment is known to increase arterial SIRT1 protein and mRNA expression in rabbits.^96^ Furthermore, increased SIRT1 mRNA levels can be detected in rabbit liver, heart, and spleen after 14 days of supplementation with nicotinic acid indicating a systemic upregulation of SIRT1 by nicotinic acid consumption. Histone acetylation levels in response to increased SIRT1 expression driven by nicotinic acid supplementation have not been studied, but it is probable that increased SIRT1 levels promote enhanced protein deacetylation. CD40 expression levels decrease in response to nicotinic acid-induced promotion of SIRT1 levels indicating the likely mechanism involves deacetylation of NF-κBp65 by SIRT1. CD40 levels are under the control of NF-κBp65, a transcription factor that can be deacetylated thereby reducing the transcriptional activation potential and reducing CD40 expression.^97^ Evidence of elevated NF-κBp65 deacetylation by SIRT1 in response to nicotinic acid treatment again demonstrates enhancement of SIRT1 activity but determining whether histone acetylation changes also follow requires direct measurement. Evidence indicates that when intracellular SIRT1 levels are reduced, histone acetylation subsequently increases, along with a concomitant increase in NF-κBp65 acetylation.^98^ Additionally, studies have demonstrated that the protein expression levels of SIRT1 have a direct negative correlation with the levels of nuclear histone acetylation which makes it plausible that nicotinic acid-induced SIRT1 expression changes alter histone acetylation levels, but this has not been shown in the context of nicotinic acid treatment specifically.^95,98–100^

Furthermore, nicotinic acid supplementation has been demonstrated to reverse depletion of SIRT1 and SIRT6 levels in primary rat aortic smooth muscle cells cultured in osteogenic media containing high phosphate to model vascular calcification.^101^ Aortic calcification was determined to be attenuated *in vivo* by nicotinic acid treatment *via* a SIRT1- and SIRT6-dependent signaling pathway. Immunoblotting detected nicotinic acid induction of SIRT1 and SIRT6 in osteogenic media, but again levels of histone acetylation were not measured in this study prompting the question of whether this upregulation of SIRT family enzymes correlates with increased histone deacetylation. Interestingly, in this study SwissTargetPrediction identified SIRT family enzymes as potential binders of nicotinic acid, and molecular docking analysis calculated binding to SIRT1 and SIRT6 with affinity equal to −5.687 kJ mol^−1^ and −5.937 kJ mol^−1^ respectively. Whether nicotinic acid can directly modulate SIRT1 and SIRT6 activity through binding is yet undetermined, but its ability to regulate levels of these critical epigenetic eraser enzymes advances the idea that this molecule can regulate acetylation-specific epigenetic mechanisms.

Additionally, like the two other vitamers, nicotinic acid can promote the depletion of the methyl donor SAM which has consequences for SAM-dependent epigenetic pathways. In rats supplemented with nicotinic acid, a significant increase in N-Me-NAM levels is observed, indicating an increase in the consumption of SAM methyl-donors.^102^ Rats administered 100 or 800 mg kg^−1^ nicotinic acid 5 times every 2 hours have levels of N-Me-NAM ∼2-fold and ∼5-fold higher respectively. SAM concentrations and methylation-specific markers were not investigated in this animal study; however, the original authors conclude that chronic high niacin exposure may “…play a role in DNA-methylation-mediated epigenetic changes…” further reinforcing the possibility of this compound's epigenetic modulating effect. This conclusion is based on the author's interpretation of their findings and requires experimental demonstration to confirm. The findings from this study are particularly interesting, because the observation that nicotinic acid stimulates the production of N-Me-NAM, a methylated nicotinamide species, indicates that nicotinic acid supplementation potentially enhances flux through NNMT-dependent pathways or increases the intracellular pool of nicotinamide. This mechanism highlights the complexity of vitamin B3 metabolism and likely has additional consequences that require further elucidation to understand how the biochemical pathways of vitamin B3 metabolism cooperate in regulating epigenetic pathways.

Finally, a novel role for nicotinic acid was recently discovered that demonstrated it exists as a bona-fide PTM on histone proteins through its covalent attachment to lysine residues, and that it contributes directly to the epigenetic control of gene expression.^103^ This novel PTM, named lysine nicotinylation (*K*_nic_), was revealed in the investigation of the nicotinic acid and nicotinamide forms of vitamin B3 in hepatocellular carcinoma. This study demonstrated that nicotinic acid suppresses the growth and metastasis of tumors whereas nicotinamide promotes the growth and metastasis. This phenomenon is linked to the existence of a novel PTM formed from nicotinic acid. Metabolism of nicotinic acid generates a highly reactive nicotinyl-CoA species (Fig. 2) that can function as a substrate for deposition of the nicotinyl moiety to proteins. Acyl-CoA species, like nicotinyl-CoA, are co-substrates for histone acetyltransferase (HAT) enzymes, critical epigenetic modifiers associated with acetylation and other short-chain fatty acylations of lysine residues on nuclear histone proteins. Acyl-CoA molecules also non-enzymatically react with lysine residues on proteins to produce the corresponding lysine acylation.^104,105^

Nicotinic acid was found in liver cells to first be converted by the action of acetyl-CoA synthetase 2 (ACSS2) to nicotinyl-CoA which then reacts with free lysine residues in a HAT-independent manner to non-enzymatically generate *K*_nic_ marks on histone proteins. *K*_nic_ marks on histones have a potent effect on chromatin architecture by significantly reducing accessibility through enhancement of chromatin compaction. Several hypo-accessible genes induced by *K*_nic_ formation are associated with tumorigenesis, potentially uncovering how nicotinic acid can suppress tumor growth. Additionally, CUT&Tag sequencing experiments find *K*_nic_ sites are preferentially associated with promoter sites of genes like TEAD3, PPFIA1, and CMC2. qPCR shows attenuated expression of these genes indicating a strong epigenetic regulating effect by *K*_nic_ marks. Reduction in PPFIA1 expression by increased *K*_nic_ levels on histones is attributable to suppression of binding by regulator homeobox B9 (HOXB9) and a subsequent reduction of hepatocellular carcinoma progression. The ability for nicotinic acid to form a PTM is unique to this vitamer, indicating this compound can specifically regulate precise mechanisms of epigenetic gene regulation by altering chromatin structure or serving as a histone marker. This mechanism opens numerous unexplored avenues to further understand metabolism-driven pathways of epigenetic regulation and the role vitamin B3 metabolism plays in this context.

Through the activation of SIRT-mediated protein deacetylation, the induction of SIRT family enzyme expression, induction of a methyl sink, and the formation of a novel epigenetic PTM on histone proteins, nicotinic acid influences several mechanisms of epigenetic gene regulation. Considerable research is required to resolve the specific sites of histone deacetylation under SIRT control that respond to nicotinic acid supplementation or depletion and how they correlate with gene expression. Additionally, since the discovery of *K*_nic_ as a *bona fide* PTM, a new avenue exists to understand metabolism-driven mechanisms of PTM and epigenetics. Functional studies to make sense of the site-specific effects of *K*_nic_ marks are essential in this pursuit that will aid in understanding essential mechanisms underlying human biology. Insight into the role of nicotinic acid metabolism and histone *K*_nic_ in cancer biology may inform the development of anti-cancer therapeutics and diagnostic measures.

### 4.5. Epigenetic influence through NAD^+^/NADH

NAD^+^, the central metabolite from vitamin B3 metabolism, is tied to epigenetic mechanisms of gene regulation through its function as a substrate for several families of epigenetic modifying enzymes. NAD^+^ is consumed by SIRT eraser enzymes and PARP writer enzymes, both of which are recognized as two chromatin modifier families that play key roles in epigenetic mechanisms as previously discussed. Likely, because all three vitamers of vitamin B3 can be metabolically converted to NAD^+^, the effects associated with supplementation or depletion of the three vitamers theoretically are shared with effects associated with NAD^+^.

As shown by experimental studies, SIRT-mediated histone deacetylation is enhanced by supplementing cells with NAD^+^ precursors, which leads to improved chromatin stability, as well as transcription of stress-response genes, a phenomenon discussed earlier.^106–108^ In studies of caloric restriction in mice, NAD^+^ levels elevate and SIRT1 activity increases in muscles with calorie restriction.^109^ Increasing NAD^+^ levels are attributed as the cause for the increasing SIRT1 activity. Likewise, NAMPT, the rate-limiting enzyme in NAD^+^ biosynthesis from nicotinamide, when overexpressed increases the activity and protein abundance of SIRT1.^110^ This indicates that increasing the intracellular pool of NAD^+^ through nicotinamide likely enhances SIRT1-mediated deacetylation. Indeed, SIRT1 activity increases attenuate p53 acetylation, evidence of increased protein deacetylation, suggesting that histones may also experience increased deacetylation by SIRT1 in response to elevated NAD^+^. Additionally, NMNAT overexpression increases NAD^+^ levels in a mouse dorsal root ganglion explant model and provides axonal protection through SIRT1-dependent pathways.^111^ Conceivably, NMNAT overexpression increases flux through this pathway, potentiating nicotinamide consumption and promoting NAD^+^ formation to drive SIRT1-catalyzed deacetylation. Again, this study did not examine changes to histone acetylation in response to NMNAT overexpression, leaving considerable questions about the exact mechanisms and whether epigenetic mechanisms play a considerable role in conferred axonal protection.

Depletion of NAD^+^ on the other hand, either through age or metabolic stress, inhibits SIRT function, which results in the hyperacetylation of histone proteins, as well as dysfunction in transcriptional regulation.^5^ Considerable evidence exists acknowledging SIRT enzymes function as a sensor of NAD^+^ levels that allow for a cell to dynamically respond to the metabolic conditions and context-dependent demands for epigenetic modulation.^112^ Indeed, the idea that SIRT1 functions as a biosensor for NAD^+^ levels is supported by the fact that estimated intracellular levels of NAD^+^ are within the *K*_m_ values of SIRT1 for NAD^+^ (*K*_m_ = 0.05–0.5 mM).^113–115^ Changes to NAD^+^ levels, like those demonstrated to occur in a circadian fashion, will likely alter the enzymatic activity of SIRT1 and downstream epigenetic mechanisms.^116,117^

With regards to DNA methylation, NAD^+^ plays a role through several means including PARP1 enzyme-mediated mechanisms as evidenced by several recent studies. The observation was made that treatment with NAD^+^ inhibits the growth of K562 cells, a myelogenous leukemia cell line, and that poly ADP-ribose (PAR) polymers accumulate as quickly as 24 hours upon treatment indicating stimulated PARP enzyme activity.^118^ Treatment with 10 mM NAD^+^ caused a 44% and 60% reduction in DNA methylation at three transcription start sites upstream of the CEBPA gene, a known methylation-sensitive gene, at 48- and 72-hours post NAD^+^ addition respectively. This reduction in DNA methylation is associated with a significant increase in mRNA levels of CEBPA. NAD^+^-induced demethylation also occurs in HEK293 and Jurkat cells, demonstrating the ubiquitous nature of this mechanism across tissue types. In HEK293 cells, both a decrease in DNA methylation and an increase in CEBPA mRNA occur, whereas in Jurkat cells no changes in mRNA levels of CEBPA occur despite even lower DNA methylation levels than in HEK293 cells with NAD^+^ treatment. Possibly, PAR polymers proximal to the promoter regions targeted by DNMT enzymes impair enzymatic activity preventing deposition of methyl groups to those sites.

Moreover, PARP1 enzymes consume a considerable fraction of NAD^+^ under certain conditions, and these enzymes regulate intracellular concentrations of DNMT1 as well.^119–121^ PARP1 specifically localizes to the promoter region of the Dnmt1 gene preventing methylation at this site and the transcriptional silencing of this gene.^122^ In doing so, the levels of DNMT1 protein are maintained, protecting normal genomic methylation patterns. DNMT1 has been demonstrated to methylate the promoter for its own gene, regulating its expression so this mechanism may prevent the silencing of itself.^123^ This effect is mediated by PARylated PARP1 and is lost in the absence of PARs suggesting the enzymatic activity of PARP1 is required to maintain normal genomic methylation patterns. Alterations to PARP1 activity through changes to NAD^+^ availability are likely to alter the levels of DNMT1 proteins and DNA methylation *via* this mechanism.

Interestingly, these findings appear to contradict the previous study where NAD^+^ treatment promoted PAR polymer formation and decreased DNA methylation at several sites.^118^ Decreased silencing of Dnmt1 by PARP1 plausibly increases DNMT1 protein levels which should stimulate global increases to DNA methylation. However, since evidence has demonstrated that PARylated PARP1 can localize to DNA promoter regions blocking methylation, like in the case of Dnmt1, the possibility arises that PARylated PARP1 can localize to other DNA sequences to block methylation and silencing at those sites. Treatment with NAD^+^ stimulates PARP1 activity and PAR formation which may induce PARylated PARP1 to localize to other promoter sites blocking their methylation. One possible explanation is that localization of PARylated PARP1 to specific genomic loci is differentially regulated by particular chromatin states and associated factors, rather than acting globally, which accounts for the discrepancy between site-specific hypomethylation and DNMT1 levels. Further experimental investigation is necessary to dissect the detailed mechanisms of PAR-mediated decreases to DNA methylation in response to NAD^+^ perturbations. Dysregulation of PARylation by alterations to NAD^+^ levels or vitamin B3 metabolism consequently may have a significant effect on DNA methylation globally.

NAD^+^ may also regulate the intracellular concentrations of DNA methyltransferase enzymes through another mechanism that permits NAD^+^ to modulate DNA methylation status. Using primary ovarian cancer cells with and without BRCA1 mutations, a relationship was identified between NAD^+^, DNMT1, and BRCA1 mutants.^124^ In non-BRCA1 mutant primary cancer cells supplemented with NAD^+^, the relative levels of BRCA1 mRNA increase with NAD^+^, and strikingly there is an almost 2.5-fold increase in DNMT1 mRNA levels. When NAMPT is overexpressed to promote NAD^+^, a corresponding increase in DNMT mRNA levels is seen in non-BRCA1 mutant cells. The opposite trend observed is with a NAMPT shRNA-mediated knockdown. Ultimately, the global levels of DNA methylation in response to NAD^+^-induced DNMT mRNA upregulation have not been examined, and the actual protein levels have not been assessed, leaving important questions unanswered.

Furthermore, SIRT1, activated by NAD^+^, deacetylates DNMT1 proteins to increase their DNA methyltransferase activity thereby altering transcriptional repression *via* this epigenetic mechanism.^125^ Treatment with EX-527, a SIRT1-specific inhibitor, in HEK293T cells results in considerable decreases to DNMT enzymatic activity. Additionally, endogenous immunopurified DNMT1 from Sirt1^−/−^ MEF cells has less enzymatic activity than that from Sirt1^+/+^ MEF cells. RNAi depletion of SIRT1 levels in HeLa cells increases acetylated DNMT1 levels ∼3-fold. Taken together, it is evident that SIRT1 regulates the acetylation of DNMT1 and the methyltransferase activity of this enzyme. Two lysine residues in the catalytic site of DNMT1 (K1349 and K1415) are the sites of acetylation that allow SIRT1 to regulate the silencing of specific tumor suppressor genes like ESR1 and CDH1 through modulation of DNMT1 methyltransferase activity. Unfortunately, the importance of the cellular NAD^+^ status in this mechanism has not been assessed, leaving a gap in our understanding of how the process is regulated by vitamin B3 metabolism.

It is important to keep in mind that these NAD^+^-consuming enzymes generate nicotinamide as a product of the reaction that can be recycled through the salvage pathway back into NAD^+^ or may serve as product inhibitors of these reactions. As previously mentioned, other NAD^+^ precursors like nicotinic acid and NR are likely to contribute to the same epigenetic mechanisms documented with NAD^+^. More work is required to determine the interplay between concentrations of NAD^+^ and the three vitamers of vitamin B3, and how flux through these metabolic pathways can regulate the epigenetic mechanisms described above in context-dependent manners.

### 4.6. Epigenetic influence through NADP^+^/NADPH

NADP^+^, and the reduced form, NADPH, are the phosphorylated forms of NAD^+^ that can be interconverted by the action of kinases and phosphatases.^126,127^ NADP^+^ and NADPH have both been identified as potent chemical modulators of several protein modifying enzyme families that are intrinsically linked to epigenetic regulation of gene expression. PTM of histone proteins by the addition of acetyl moieties to the ε-amino group of lysine residues represents a fundamental epigenetic mechanism that cells employ to dictate gene transcription by modulating relative DNA accessibility at sites proximal to acetylation.^9^ Increases to histone acetylation induce gene transcription by relaxing chromatin compaction at the sites of modification thereby promoting transcription factor binding and recruitment of adaptor proteins to modification sites.^128^ This process is dynamically controlled by histone acetyltransferases enzymes (HATs) that deposit the acetyl moiety and histone deacetylase enzymes (HDACs) and SIRTs that remove the acetyl moiety.^129^ Modulation of HAT or HDAC/SIRT activity has dramatic consequences for transcription and gene expression which has driven considerable investigation into regulation of HDAC/SIRT activity and the development of anti-cancer therapeutics that target HDACs/SIRTs.^130^ NADP^+^ and NADPH have been reported as potent modifiers of these mechanisms that will be explored here.

Divided into three classes (class I, II, and IV), HDAC enzymes are distinct from the closely related family of SIRT deacetylase enzymes that similarly remove acetyl marks from lysine residues as previously discussed. HDAC3, a class I HDAC, is unique from the other class I HDACs (HDAC1, HDAC2, and HDAC8) as it is enzymatically inactive unless bound by its partner, nuclear receptor corepressor (NCOR), to form an HDAC3–NCOR complex that exhibits deacetylase activity.^131^ NADPH was recently identified as an endogenous inhibitor of HDAC3 activity through direct interaction that disrupts the HDAC3–NCOR complex.^132^ Interestingly, surface plasmon resonance experiments demonstrated that NADPH exclusively could bind to HDAC3 with *K*_D_ = 1.81 µM, whereas the structurally similar NADP^+^, NAD^+^, and NADH had either reduced or no binding. The unique ability for NADPH to inhibit this complex, but not NADP^+^ implies some form of regulation in cells that depends on the NADP^+^/NADPH ratio which is linked to a cell's overall reductive potential. NADPH inhibits HDAC3 in a dose-dependent manner and in 3T3-L1 preadipocytes increases global histone acetylation in a dose-dependent manner, indicating a significant epigenetic modulating effect. Critically, this mechanism is required for adipocyte differentiation as well, demonstrating the potency of NADPH's epigenetic modulating effect and the functional outcomes. This mechanism of HDAC3 inhibition may be an important but unappreciated mechanism that requires further investigation as evidenced by its widespread impacts on global histone acetylation and cell differentiation.

On the other hand, NADPH is an activator of the deacetylase activity of HDAC1 and HDAC2 as measured *in vitro* by radiometric scintillation assay.^133^ A ∼2-fold increase in HDAC activity is observed when NADPH is added to an HDAC-catalyzed reaction; however, similar to NADPH's ability to inhibit HDAC3, this interaction was exclusive to NADPH and not NADP^+^, NAD^+^, or NADH. NADPH enhances HDAC1 and HDAC2 activity through direct interaction, increasing the velocity of the reaction (*V*_max_) with a simultaneous slight decrease to the affinity (*K*_m_) of the enzyme for its substrates. The reduced phosphate group clearly specifies the interactions and indicates that these processes are tightly regulated by NADPH concentrations within the cell and some relationship with the cell's overall reductive potential exists.

When glucose-6-phosphate dehydrogenase (G6PD) is inhibited by 5-dehydroepiandrosterone (DHEA) to reduce cellular NADPH, an increase in several H4 and H3 acetylation markers like H4K8ac, H4k12ac, H4k16ac, H3K9ac, H3K14ac, and H3k18ac is observed likely due to reduced HDAC activity. This observation implies that perturbations to NADPH homeostasis within cells may have important implications regarding gene expression, as the identified acetylation sites are typically considered gene activators through increasing DNA accessibility at promoter sites within the genome.^134–136^ The opposing activation and inhibition of different HDAC enzymes by the same compound poses an interesting dilemma that requires carefully designed experiments to understand the precise epigenetic responses to NADPH and perturbations to its metabolism. Several pathways exist that contribute to the NADPH pool within cells: the phosphorylation of NAD^+^ by NAD kinase, the pentose phosphate pathway, pyruvate–malate shuttle cycle, and several other redox reactions.^126,137,138^ Not only that, but NADPH is consumed through fatty acid biosynthesis, cholesterol synthesis, and steroid synthesis indicating an important linkage between cellular metabolism, NADPH homeostasis, and acetylation-specific epigenetic regulation of gene expression.

Recent experiments revealed that NADP^+^ is an endogenous inhibitor of another family of enzymes: the PARP enzyme family. Initial *in vitro* experiments demonstrated NADP^+^ binds PARP1, PARP2, and PARP10 and *via* isothermal titration calorimetry demonstrated NADP^+^ strongly interacts with PARP1 with *K*_D_ = 39 µM.^139^ Furthermore, NADP^+^ is a dose-dependent inhibitor *in vitro* of PARP1, PARP2 and PARP10 catalyzed PARylation. Upregulation of NADP^+^ production *in cellulo* through NADK overexpression raises the NADP^+^/NAD^+^ ratio and decreases global PARylation through PARP inhibition. Histone MARylation and PARylation have not been specifically examined with alterations to intracellular NADP^+^ concentration, but global decreases in PARylation indicate likely decreases in histone PARylation as well. Direct and indirect epigenetic effects from PARP inhibition are discussed further in this review (see Section 4.8), so no further discussion is added here regarding NADP^+^ and PARP inhibition.

Ultimately, taken together with the previous sections, the relationship between NADP^+^/NADPH homeostasis within cells and the epigenetic regulation of gene expression is a highly complex process regulated by the activity of multiple pathways ubiquitous throughout the cell. Perturbations to the NADP^+^/NADPH ratio will likely lead to an altered epigenetic state by inhibition/activation of several HDAC and PARP enzymes known to be critical epigenetic regulators.

### 4.7. Epigenetic influence through *N*-methylnicotinamide (N-Me-NAM)

*N*-Methylnicotinamide (N-Me-NAM) remains a relatively understudied biomolecule due to its positioning as an intermediate generated through an excretion pathway; however, it has recently garnered some attention regarding its potential to regulate epigenetic mechanisms of gene expression. As previously discussed, NNMT is an essential enzyme that generates N-Me-NAM through the consumption of a molecule of SAM to methylate nicotinamide. This mechanism can cause methyl-donor depletion which has significant epigenetic consequences; however, this is not the only potential consequence of this enzyme's activity. The product, N-Me-NAM, has been observed to regulate the activity of multiple SIRT enzymes through two different mechanisms.

First, NNMT is able to regulate SIRT1 stability through N-Me-NAM. Treatment of N-Me-NAM in hepatocytes promotes a dose-dependent increase in SIRT1 protein expression.^140^ No increase in SIRT1 mRNA levels is detected with N-Me-NAM administration, but SIRT1 ubiquitination significantly decreases implying that N-Me-NAM increases the stability of SIRT1 enzymes by reducing degradation. Histone acetylation changes in response to SIRT1 stabilization by N-Me-NAM have not been measured. In mouse liver following N-Me-NAM supplementation, SIRT1 expression is increased and FOXO1 acetylation, a primary target of SIRT1, is reduced indicating that the observed SIRT1 stabilization enhances its protein deacetylation activity. Evidence of enhanced stability and activity in this critical epigenetic enzyme induced by N-Me-NAM treatment highlights the possibility for this biomolecule to have robust effects on epigenetic mechanisms. As previously mentioned, several studies have demonstrated that the protein expression levels of SIRT1 are directly related to levels of histone acetylation indicating that the effect observed here likely impacts histone acetylation.

Second, N-Me-NAM was found to have protective effects against high fat diet- and age-induced hearing loss in mice with the mechanism linked to SIRT1 and SIRT3 protein level elevation.^141^ Miwa *et al.* (2021) demonstrated in a mouse model that high-fat diets and aging lead to decreases in cochlear SIRT1 and SIRT3 levels. This reduction in SIRT1 and SIRT3 is reversible by supplementation with N-Me-NAM. While N-Me-NAM is unable to fully restore SIRT levels to that of young mice, the partial restoration demonstrates the ability for this biomolecule to alter levels of important epigenetic modifying enzymes. Increases in SIRT1 and SIRT3 protein levels are not due to changes in the mRNA expression as measured by qRT-PCR suggesting that the ability for N-Me-NAM to regulate SIRT expression is likely due to increased stabilization, but this has yet to be confirmed. The stabilization of SIRT1 and SIRT3 levels by N-Me-NAM having a protective influence against high fat diet- and age-induced hearing loss indicates a potent pharmacological effect.

These two studies did not investigate the effects of N-Me-NAM on histone acetylation levels, but FOXO1 acetylation was attenuated indicating that N-Me-NAM enhances protein deacetylation including the possibility for enhanced histone deacetylation. Additionally, several studies cited throughout this review showcase clearly that changes to SIRT protein levels alter histone acetylation levels providing more evidence that N-Me-NAM may modulate epigenetic mechanisms. How specifically histone acetylation is affected by N-Me-NAM through SIRT-mediated pathways remains unanswered and represents an important area for future investigation, given the accumulation of this metabolite in ageing and chronic disease.

### 4.8. Epigenetic influence through *N*-methyl-3-carboxamide-6-pyridone (N-Me-6PY)

The vitamin B3 catabolite, *N*-methyl-3-carboxamide-6-pyridone (N-Me-6PY), is one of the major end products from vitamin B3 metabolism and is often used as a measure for niacin nutritional status by quantifying its urinary excretion.^142^ While predominantly understood as a uremic toxin produced for excretion, N-Me-6PY exists within human plasma and its concentration has been demonstrated to increase with age to as much as 2.6 times higher in older humans.^143^ Interestingly, N-Me-6PY concentrations elevate dramatically in patients with chronic kidney disease (CKD) to as a high as 3100 ng mL^−1^ compared to healthy individuals with approximately 140 ng mL^−1^. Furthermore, when supplemented with nicotinamide, chronic kidney disease patients reach as high as 21 290 ng mL^−1^ N-Me-6PY suggesting significant accumulation of this compound in certain conditions, especially when kidney filtration is impaired.^46^

N-Me-6PY is a potent inhibitor of the poly(ADP-ribose) polymerase 1 (PARP1) enzyme reaction *in vitro*. N-Me-6PY bears structural similarity with known PARP1 inhibitors and dose-dependent inhibition studies with N-Me-6PY show significant inhibition at just 30 µM in biochemical assay.^144^ In advanced renal failure or CKD, serum concentrations of N-Me-6PY are high enough to inhibit PARP1 activity. This was confirmed in studies of children with chronic renal failure where plasma concentrations of N-Me-6PY reached as high as 32.5 µM, an increase as much as 32-fold compared to healthy children.^145^ In this study the IC_50_ of N-Me-6PY towards PARP1 was measured at ∼2.2 mM; however, the authors state that the measured plasma concentrations in chronic renal failure are still high enough to inhibit PARP1 activity.

In another study, the IC_50_ was measured at 8 µM, a much lower IC_50_ value that falls within the range for chronic renal failure and would lead to significant inhibition of PARP1.^46^ Despite the reported differences in the IC_50_ value of N-Me-6PY towards PARP1 from several research groups it nonetheless is evident that this catabolite from vitamin B3 metabolism can inhibit the enzymatic activity of PARP1, the most prevalent isoform in humans. Further biochemical and *in vitro* inhibition studies are required to accurately assess the IC_50_ of N-Me-6PY towards PARP1. Additionally, mechanistic studies are still required to understand the mechanism of inhibition by N-Me-6PY and *in cellulo* or *in vivo* experiments are required to document the specific alterations to PARP1-catalyzed protein MARylation and PARylation in response to elevated N-Me-6PY levels.

PARP1 predominantly localizes to the nucleus where it functions to coordinate DNA damage response processes through the covalent attachment of ADP-ribose units to histone and non-histone proteins. PARP1 can PARylate each of the core histone proteins (H2A, H2B, H3, and H4) and the linker H1 histone protein at several sites to orchestrate numerous epigenetic events.^146^ Histone PARylation catalyzed by PARP1 is crucial for the maintenance of genomic stability, chromatin condensation states, and subsequently gene expression. PARP1 modification of histones plays a role in several epigenetic mechanisms crucial to maintaining patterns of gene regulation and we can infer that inhibition of PARP1 by N-Me-6PY will inhibit these mechanisms that rely on PARylated histones.

Direct effects from N-Me-6PY inhibition of PARP1 are understudied with some studies suggesting deleterious effects and others suggesting beneficial effects depending on the context; however, studies on direct epigenetic effects of PARP1 inhibition by N-Me-6PY are non-existent in the literature reviewed, representing a significant gap in our understanding of this relationship.^145,147,148^ We can infer from other inhibition studies of PARP1 the epigenetic effects of inhibition by N-Me-6PY as this compound is reported as a potent inhibitor. Synthetic PARP1 inhibitors, PJ-34 and ABT-888, cause global decreases in histone H3 and H4 acetylation along with a reduction in mRNA levels for several histone acetyltransferases like p300 and PCAF in several cell lines.^149^ PARP1 inhibition also promotes a global increase in HDAC activity towards histone H3 with no change to HAT activity. This suggests that PARP1 inhibition can promote histone hypoacetylation through activation of HDAC enzymes. Interestingly, a select number of histone lysine residues increase in acetylation despite global acetylation decreases raising the possibility that inhibition of PARP1 activity frees lysine sites for acetylation that would otherwise be blocked by PAR polymers. Acetylation-specific epigenetic marks are clearly affected by inhibition of PARP1 thus it goes without saying that increasing intracellular N-Me-6PY concentration may elicit similar epigenetic effects.

Beyond histone substrates, PARP1 regulates several epigenetic mechanisms through its writer activity. PARP1 PARylates the histone lysine demethylase enzyme KDM5B which leads to inhibition of KDM5B, an increase in H3K4me3 at promoter sites, and the exclusion of linker histone H1.^150^ This process drives an increase in transcription start site accessibility and loading of RNA Pol II machinery to promote gene transcription. Inhibition of PARP1 by N-Me-6PY may activate KDM5B, leading to increased lysine demethylation and the promotion of a hypomethylated chromatin state that attenuates gene transcription at sites proximal to demethylation events by preventing RNA pol loading. PARP1-catalyzed PARylation of NSD2, a lysine methyltransferase that specifically deposits dimethylation marks to histone H3K36, leads to significant inhibition of the NSD2 enzyme and a decrease in H3K36me2 marks.^151^ Inhibition of NSD2 PARylation may activate this enzyme leading to hypermethylation at H3K36.

In addition to lysine methyltransferases and demethylases, PARP1 has been found to PARylate the lysine acetyltransferase, p300, which stimulates its activity and promotes histone acetylation thereby driving a multitude of downstream epigenetic effects through the stimulation of p300-dependent gene transcription.^152^ p300 is regarded as the predominant acetyltransferase in human cells and is responsible for as much as 1/3 of the nuclear acetylome making p300 one of the most important epigenetic modifying enzymes in human biology.^153^ Inhibition of PARP1 by N-Me-6PY could diminish p300 PARylation and subsequently reduce p300 acetyltransferase activity thereby altering the global profile of histone acetylation and gene expression. This may cooperate with the previously mentioned process where PARP1 inhibition attenuates PCAF and p300 mRNA levels to drive an even further reduction in histone acetylation levels.

Beyond PTM of histones, PARP1 inhibition also affects DNA methylation status. PARP1 directly regulates DNA methylation through a proposed mechanism whereby PARylated PARP1 physically interacts with DNMT1 enzymes. This noncovalent interaction between PARP1 and DNMT1 prevents DNMT1 from binding to DNA, ultimately blocking the methylating activity of this enzyme and driving a hypomethylated state in cells.^154^ Indications of this phenomenon were observed through experiments that found PARP inhibition promotes DNA hypermethylation *in vivo*, prompting investigation into this relationship. In biochemical assay format with PARP1, DNMT1, free DNA, and the necessary reaction substrates, treatment with DPQ, a PARP1 inhibitor, increased methyl incorporation by DNMT1 onto DNA by 2.5-fold compared to control confirming that PARP1 indirectly regulates DNA methylation. Incubation of PARylated PARP1 with DNMT1 causes a methyl incorporation decrease of ∼50–60% indicating a clear inhibitory effect by PARylated PARP1. Inhibition of PARP1 activity by N-Me-6PY can plausibly inhibit the PARylation of PARP1 thereby preventing the physical association of PARP1 and DNMT leading to increased DNA methylation as seen with DPQ. These findings point to a role for N-Me-6PY in DNA methylation and that the prevalence of DNA methylation marks is intertwined with PARP1. Possibly N-Me-6PY status within cells can regulate DNA methylation through PARP1. Functional studies to document and understand the specific loci regulated by this process are essential to understand the epigenetic ramifications, and further studies with N-Me-6PY are required to confirm this effect can be recapitulated with this compound.

To further the possible role for N-Me-6PY in epigenetic mechanisms of gene regulation relating to DNA methylation, inhibition of PARP1 activity by the synthetic inhibitors PJ-34 and ABT-888 decrease in 5-hydroxymethylcytosine (5hmC) levels in HEK293T cells.^155^ 5hmC, formed by the oxidation of 5-methylcytosine (5mC), was reported in 2009 and quickly recognized as a critical epigenetic mark on DNA with unique biological functions in transcriptional regulation leading to its designation as the “sixth base”.^156–158^ Reduction in 5hmC levels by PARP1 inhibition is due to a decrease in PARylation of the TET1 hydrolase enzyme that acts to convert 5mC into 5hmC. Covalent PARylation of TET1 activates the enzyme, so, PARP1 inhibition by PJ-34 and ABT-888 attenuates PARylation of TET1 blocking its ability to oxidize 5mC. The observation that PARP1 inhibition leads to alterations in 5hmC levels in cells demonstrates another potential mechanism through which N-Me-6PY can alter the epigenetic mechanisms of gene expression. If N-Me-6PY inhibits PARP1 PARylation of TET1 it is likely that 5mC marks accumulate when N-Me-6PY levels are elevated in states such as in renal failure or CKD which will drive a hypermethylated state.

While the role of N-Me-6PY in epigenetic mechanisms is not clearly defined, we can infer from the studies presented above that N-Me-6PY is likely to play a significant role in histone acetylation, DNA methylation, and downstream gene expression patterns. As a potent inhibitor of PARP1, N-Me-6PY likely affects the same mechanisms understood to be modulated by other synthetic PARP1 inhibitors. Understanding how vitamin B3 metabolism can affect these mechanisms is still unclear as the role of N-Me-6PY is undefined in the already performed studies.

## 5. Summary and perspectives

Vitamin B3 metabolism generates a diverse array of metabolites (Fig. 2) that are essential for maintaining homeostasis and normal physiological function; however, the roles of vitamin B3 and its metabolites in epigenetic mechanisms of gene regulation are just now being appreciated. Owing to the structural diversity of vitamin B3 and its metabolites, their complex intertwining networks, and the cell- or tissue-specific metabolic demands, epigenetic regulation mechanisms by vitamin B3 metabolites are exceedingly complex. Some biomolecules like nicotinamide, NAD^+^, and NADP^+^ have clearly defined direct roles through their ability to serve as inhibitors or substrates for epigenetic modifying enzymes. On the other hand, some of these and others discussed above have more indirect effects that are less defined and require additional experimental evidence to confirm. Overall, vitamin B3 metabolites appear to impact epigenetics through two fundamental biochemical mechanisms: reversible binding with and regulation of epigenetic-controlling proteins or enzymes, and covalent modification of nuclear DNA, histones, and other chromatin regulating proteins (Table 2). Regulation of RNA biology by vitamin B3 metabolites is also highly possible but warrants experimental investigation (Fig. 3–8).^159^

**Table 2:** 

Metabolite	Targeted/affected enzymes	Epigenetic mechanisms/regulated pathways
Nicotinamide	(1) SIRT1–7	(1) Inhibition of SIRT1–7 enzymatic activity and attenuation of histone deacetylation^65–67^
	(2) PARP1	(2) Inhibition of PARP1 enzymatic activity and attenuation of histone MARylation and PARylation^68–70^
	(3) NNMT	(3) Activation of NNMT enzymatic activity and attenuation of histone methylation. Attenuation of DNA methylation (methyl-sink)^79–85^
Nicotinamide riboside	(1) NNMT	(1) Activation of NNMT enzymatic activity and attenuation of DNA methylation (methyl-sink)^90,91^
	(2) DNMT3l/3b	(2) Attenuation of DNMT3l/3b gene expression and reduction in DNA methylation^91^
	(3) TET3	(3) Induction of TET3 expression and attenuation of DNA methylation^91^
	(4) SIRT1 and SIRT3	(4) Activation of SIRT1 and SIRT3 enzymatic activity and attenuation of FOXO1 acetylation. Induction of histone deacetylation?^92^
	(5) SIRT5	(5) Allosteric activation of SIRT5 enzymatic activity. Enhanced histone deacetylation^93^
Nicotinic acid	(1) SIRT1	(1) Activation of SIRT1 enzymatic activity, induction of SIRT1 protein and mRNA expression, and attenuation of p53 acetylation. Induction of histone deacetylation?^94–96,101^
	(2) SIRT6	(2) Induction of SIRT6 expression. Induction of histone deacetylation?^101^
	(3) NNMT	(3) Activation of NNMT enzymatic activity (methyl-sink). Enhancement of N-Me-NAM intracellular concentration. Attenuation of histone and DNA methylation?^102^
	(4) ACSS2	(4) Formation of nicotinyl-CoA and post-translational modification of histones with lysine nicotinylation (*K*_nic_)^103^
NAD^+^	(1) SIRT1–7	(1) Activation of SIRT1–7 enzymatic activity and induction of p53 deacetylation. Induction of histone deacetylation?^106–114^
	(2) PARP1	(2) Activation of PARP1 enzymatic activity and induction of PAR formation. Reduction in DNA methylation^117^
	(3) DNMT1	(3) Activation of SIRT1 enzymatic activity induces deacetylation of DNMT1 and DNA methylation. Induction of DNMT1 expression^118–124^
NADP^+^	(1) ARP1, PARP2, and PARP10	(1) Inhibition of PARP1, 2, and 10 enzymatic activity and attenuation of global PARylation. Attenuation of histone PARylation?^139^
NADPH	(1) HDAC1 and HDAC2	(1) Activation of HDAC1 and HDAC2 enzymatic activity. Induction of histone deacetylation.^133^
	(2) HDAC3	(2) Disruption of HDAC3–Ncor complex and inhibition of HDAC3 enzymatic activity. Attenuation of histone deacetylation^132^
*N*-Methylnicotinamide (N-Me-NAM)	(1) SIRT1	(1) Reduction in SIRT1 ubiquitination and induction of SIRT1 protein levels. Attenuation of FOXO1 acetylation. Induction of histone deacetylation?^139,140^
	(2) SIRT3	(2) Induction of SIRT3 protein levels. Induction of histone deacetylation?^140^
*N*-Methyl-3-carboxamide-6-pyridone (N-Me-6PY)	(1) PARP1	(1) Inhibition of PARP1 enzymatic activity and PAR formation.^143,144^
	(a) HDAC?	(a) PARP1 inhibition enhances HDAC activity?^148^
	(b) KDM5B?	(b) PARP1 inhibition enhances histone methylation?^149^
	(c) NSD2?	(c) PARP1 inhibition enhances histone demethylation?^150^
	(d) p300?	(d) PARP1 inhibition inhibits histone acetylation?^151^
	(e) DNMT1?	(e) PARP1 inhibition enhances DNA methylation?^153^
	(f) TET1?	(f) PARP1 inhibition enhances DNA methylation?^154^

**Fig. 3:**
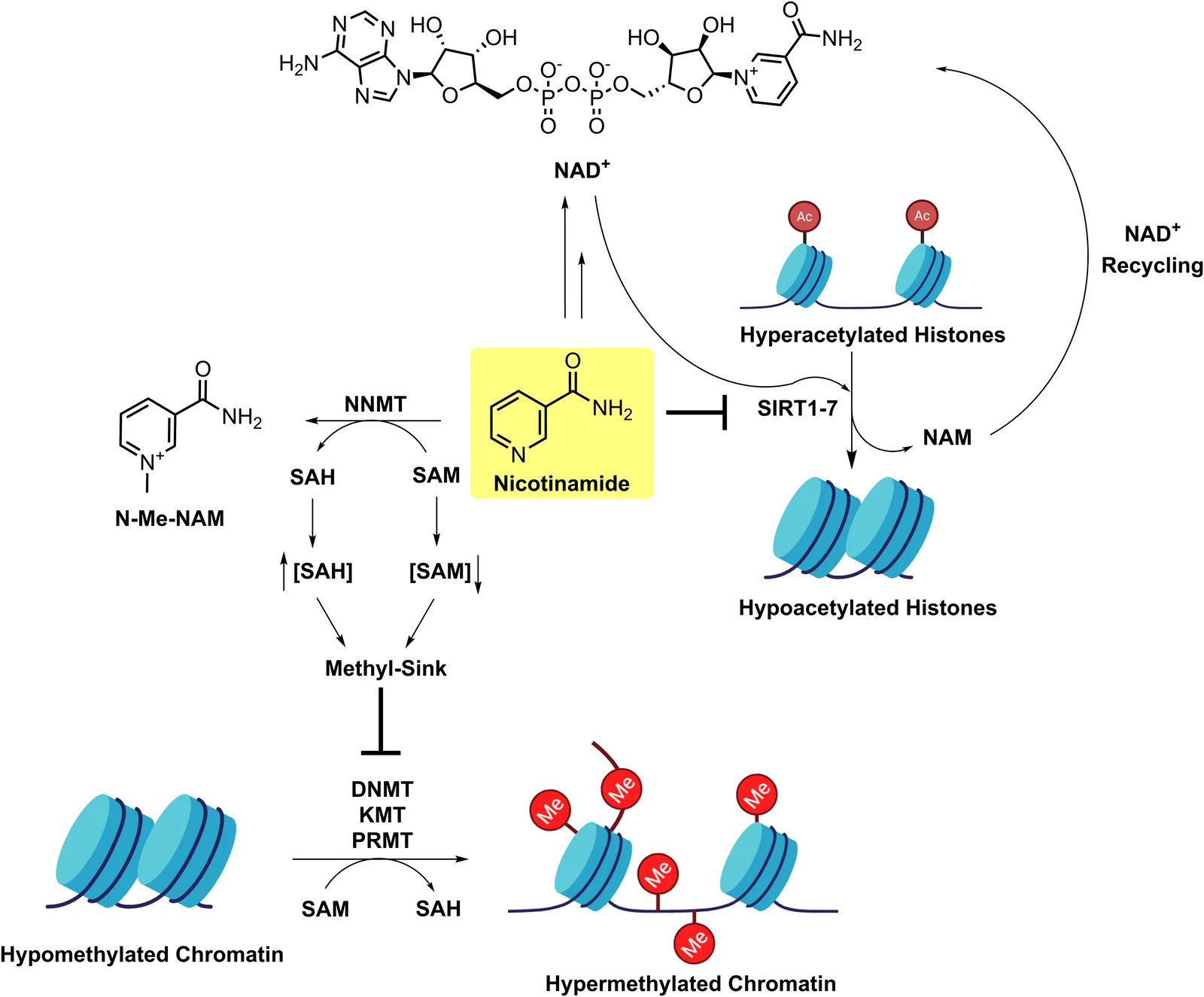
Epigenetic pathways modulated by nicotinamide. Nicotinamide (NAM) acts as an inhibitor of the SIRT-catalyzed deacetylation reaction to promote a hyperacetylated histone state by inhibiting histone deacetylation. NAM is metabolized into NAD^+^ to serve as a substrate for the SIRT-catalyzed deacetylation reaction promoting hypoacetylation of histones. The SIRT reaction produces NAM that can be recycled to NAD^+^*via* the salvage pathway. Methylation of NAM by NNMT serves as a “methyl-sink” depleting the cellular pool of SAM. Depletion of the cellular pool of SAM limits DNMT, PRMT, and KMT enzyme-catalyzed reactions preventing the formation of hypermethylated chromatin.

**Fig. 4:**
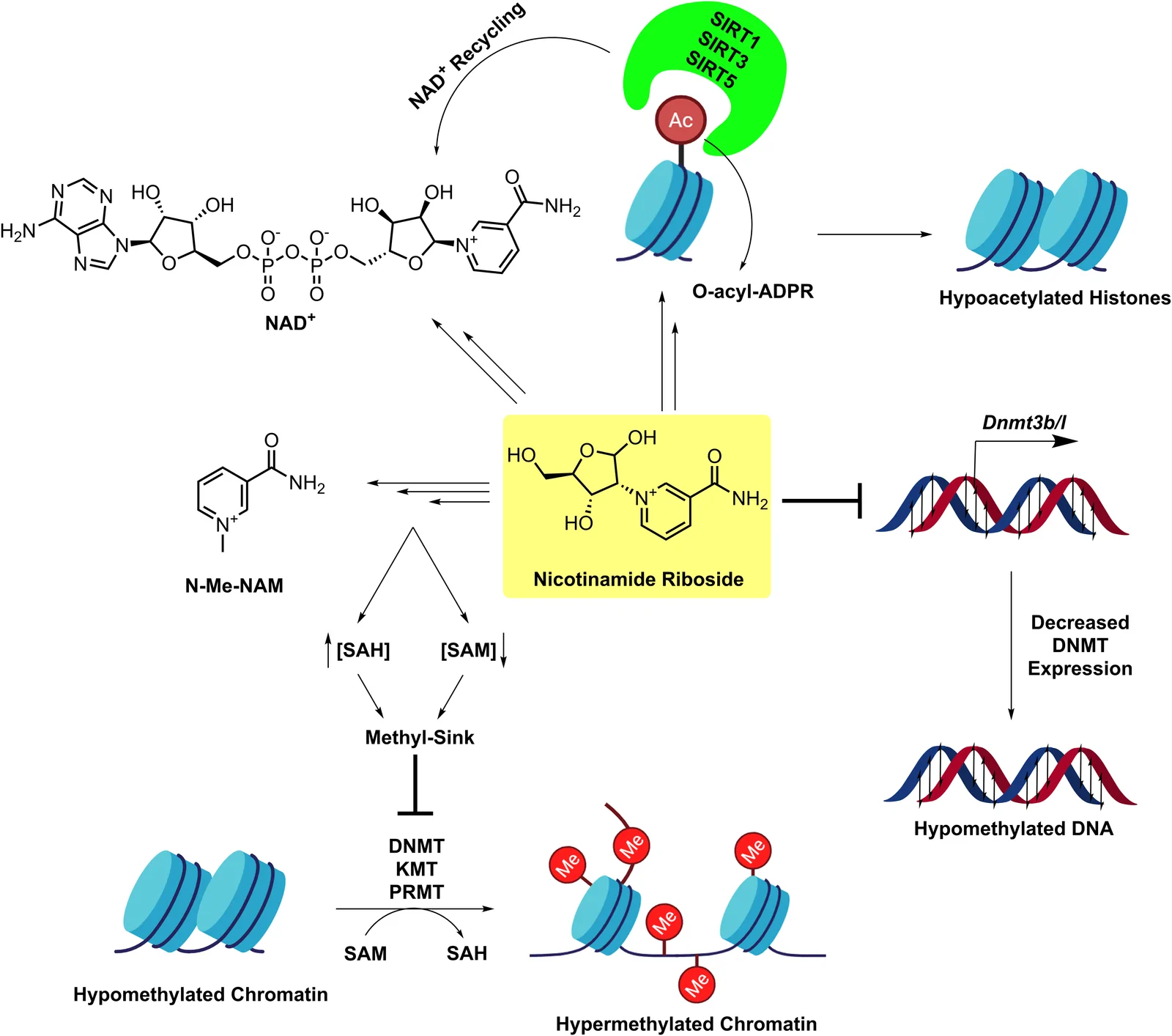
Epigenetic pathways modulated by nicotinamide riboside. Nicotinamide riboside (NR) is metabolized into N-Me-NAM, consuming SAM in the process and depleting the cellular pool of SAM. Depletion of the cellular pool of SAM limits DNMT, PRMT, and KMT enzyme-catalyzed reactions preventing the formation of hypermethylated chromatin. NR inhibits expression of Dnmt3b and Dnmt3l which leads to the promotion of DNA hypomethylation due to reduced DNA methyltransferase expression. NR supplementation activates SIRT1, SIRT3, and SIRT5 promoting protein deacetylation and potential hypoacetylation of histones. NR is metabolized into NAD^+^ and utilized for several epigenetic mechanisms reviewed elsewhere.

**Fig. 5:**
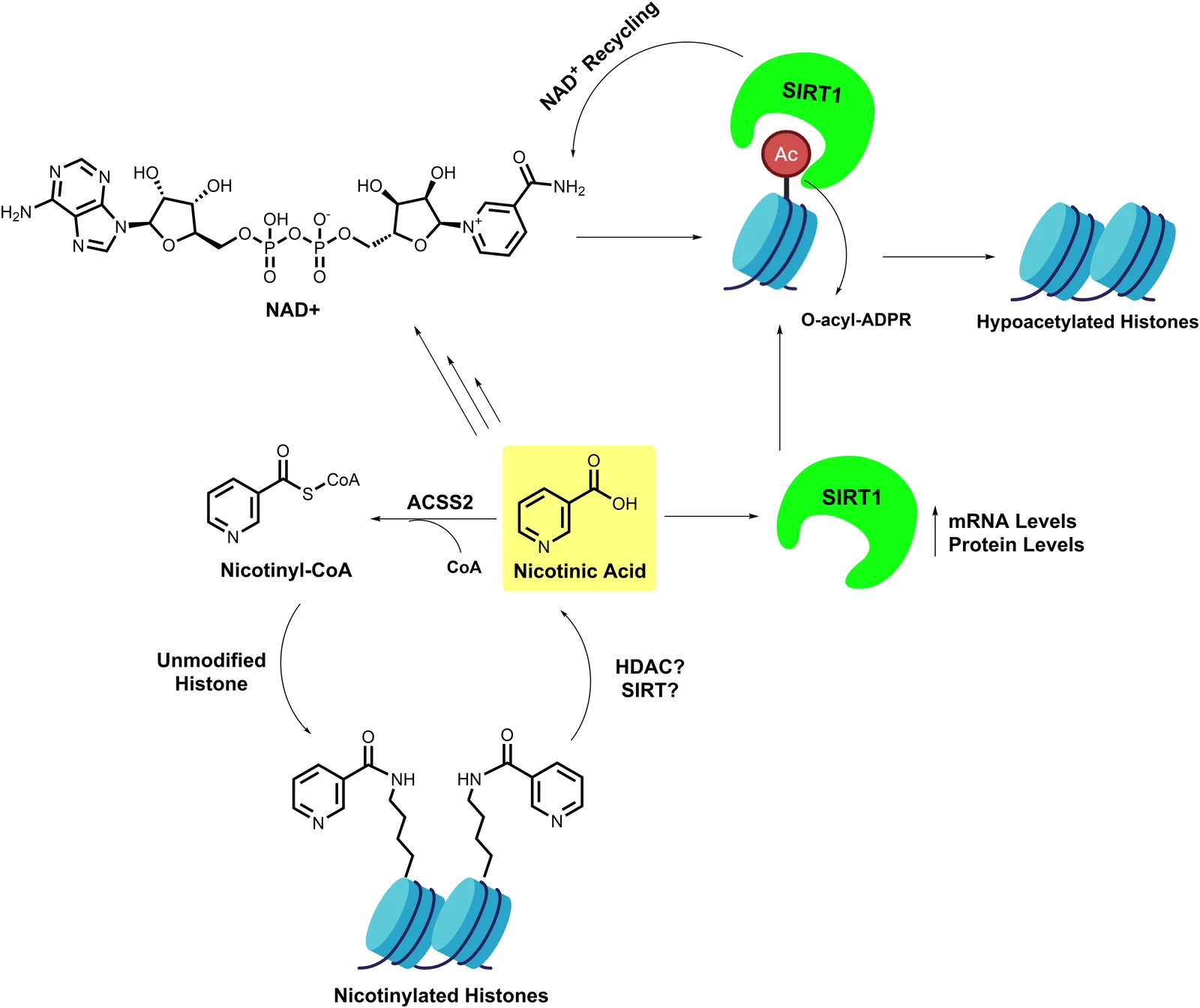
Epigenetic pathways modulated by nicotinic acid. Nicotinic acid upregulates the mRNA and protein expression levels of SIRT1, potentially promoting histone deacetylation. Nicotinic acid is metabolized into NAD^+^ to serve as a substrate for the SIRT1-catalyzed deacetylation reaction promoting a hypoacetylated histone state and producing nicotinamide. Nicotinamide can be recycled to NAD^+^*via* the salvage pathway and utilized for several epigenetic mechanisms reviewed elsewhere. Nicotinic acid can be conjugated to coenzyme A (CoA) by ACSS2 enzymes to produce nicotinyl-CoA. Nicotinyl-CoA can non-enzymatically react with free lysine residues on nuclear histone proteins to post-translationally modify them generating lysine nicotinylation and altering chromatin structure. This process is theoretically reversible by HDAC or SIRT enzymes.

**Fig. 6:**
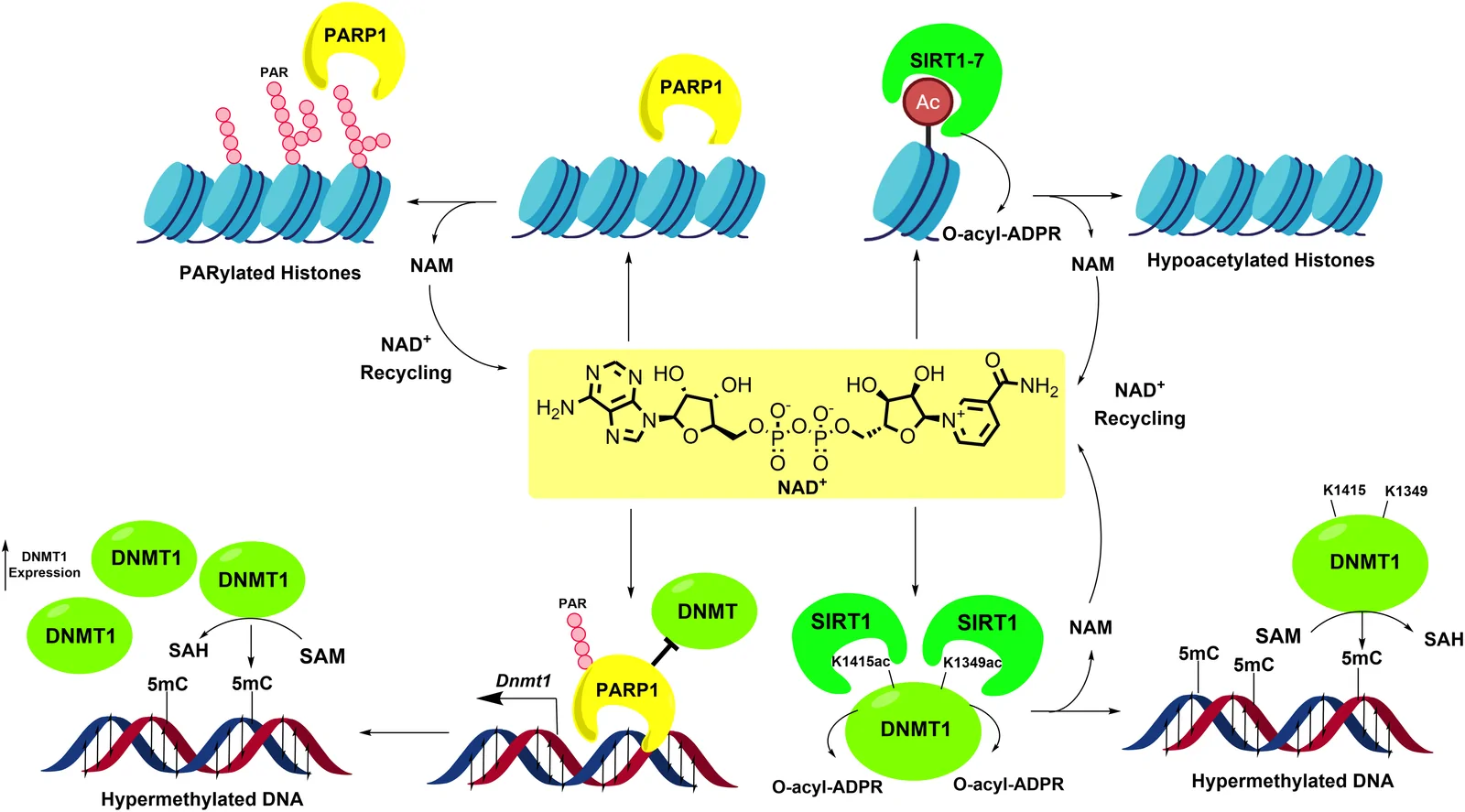
Epigenetic pathways modulated by NAD^+^. NAD^+^ is a substrate for the SIRT-catalyzed deacetylation of histone proteins. Nicotinamide (NAM) generated from this reaction can be recycled into NAD^+^*via* the salvage pathway. NAD^+^ is a substrate for PARP1 which PARylates histone proteins to orchestrate several epigenetic events relating to DNA-damage response and chromatin remodeling. NAM generated from this reaction can be recycled into NAD^+^*via* the salvage pathway. NAD^+^ activates SIRT1 enzymes to deacetylate DNMT1 at K1415 and K1349, activating DNMT1 to promote a hypermethylated DNA state. NAD^+^ activates PARP1 to promote the PARylation of PARP1 enzymes. PARylated PARP1 associates with DNA to physically block methylation of the Dnmt1 promoter to prevent its silencing and maintain normal genomic methylation by sustaining DNMT1 protein levels.

**Fig. 7:**
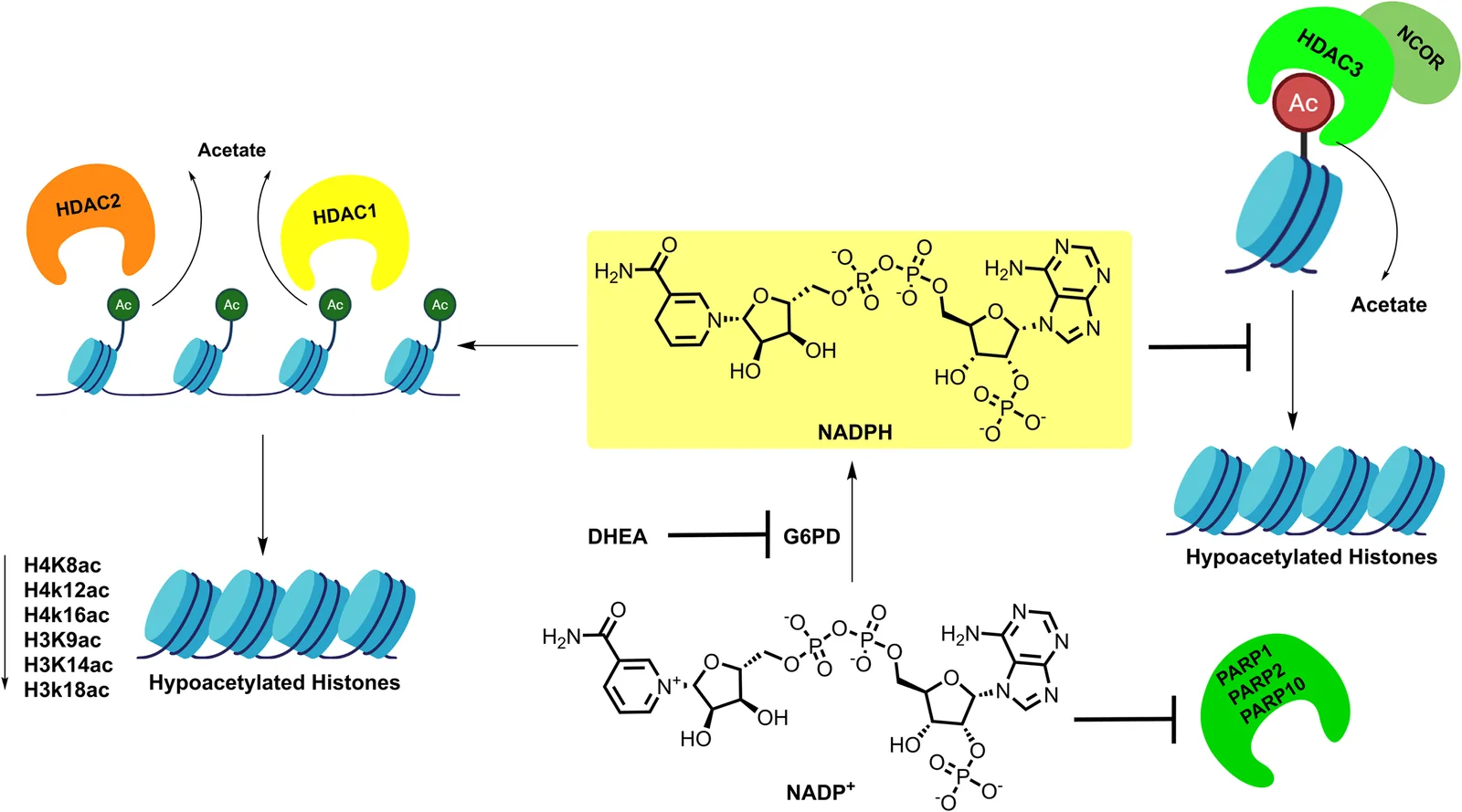
Epigenetic pathways modulated by NADP^+^/NADPH. NADPH binds to and activates HDAC1 and HDAC2 promoting a global reduction in histone acetylation. Glucose-6-phosphate dehydrogenase inhibition by DHEA reduces NADPH production leading to reduction in HDAC1 and HDAC2 activity and an increase in histone acetylation. NADPH binds to and disrupts the HDAC3–Ncor complex thereby abolishing the deacetylase activity of HDAC3 enzymes preventing the formation of a hypoacetylated histone state. NADP^+^ inhibits PARP1, PARP2, and PARP10 culminating in several downstream effects reviewed elsewhere.

**Fig. 8:**
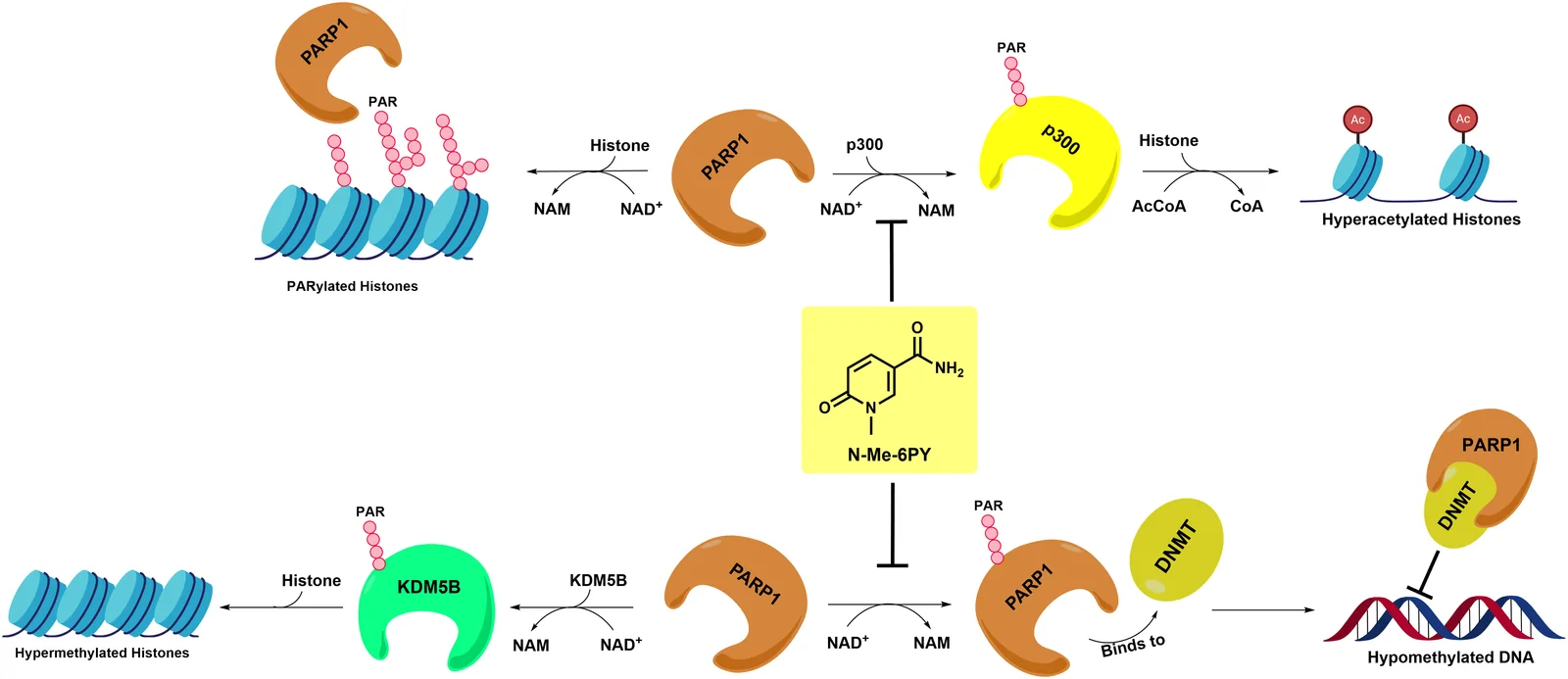
Possible epigenetic pathways modulated by N-Me-6PY. N-Me-6PY inhibits PARP1-catalyzed reactions. N-Me-6PY inhibits PARP1-catalyzed PARylation of PARP1 preventing the binding of DNMT to PARP1 and the resulting DNA hypomethylation. N-Me-6PY inhibits the PARP1-catalyzed PARylation of p300 that reduces p300's acetylation activity and potentially prevents the formation of a hyperacetylated histone state. N-Me-6PY inhibition of PARP1 inhibits histone PARylation by PARP1. N-Me-6PY inhibits PARP1-catalyzed PARylation of KDM5B that inhibits its activity thereby preventing the hypermethylation of histones.

Regarding the epigenetic effects of nicotinamide and NR, DNA methylation was extensively tested, and histone deacetylation was tested to a lesser extent. Whether other epigenetic marks, like histone lysine and arginine methylation, are affected needs to be tested. Pool depletion of the methyl donor SAM, by nicotinic acid, nicotinamide, and NR, will plausibly affect any SAM-dependent epigenetic mechanism such as histone lysine and arginine methylation. The ability of nicotinic acid to generate a novel PTM is an exciting indication that vitamin B3 plays direct roles in epigenetic mechanisms that demand further investigation to elucidate the functional importance of this newly discovered PTM. This discovery elicits a new axis to understand metabolism-driven regulation of epigenetic mechanisms through vitamins. Furthermore, as evidenced by the cited studies, several end products of vitamin B3 catabolism like N-Me-NAM and N-Me-6PY play a role in epigenetic mechanisms of gene regulation redefining these molecules as more than just waste products. These metabolites become particularly relevant during impaired kidney function and may contribute to symptomology or further disease progression through epigenetic mechanisms. Further studies should seek to examine the specific functional relationship between these vitamin B3 metabolites and the discussed epigenetic mechanisms.

The impacts of vitamin B3 metabolites on epigenetics could be time-, space-, or context-dependent. Considerable questions remain unresolved regarding how the discussed pathways interact to coordinate complex epigenetic changes that occur in response to a cell's environment. For instance, although nicotinamide acts as a product inhibitor of SIRT enzymes, feeding cells with nicotinamide will ultimately promote NAD^+^ production and subsequently enhance SIRT activity. Whether this is true for all or just some cells and the extent of this effect is still undetermined. Additionally, considerations regarding experimental design can impact the observed effects from vitamin B3 metabolism on epigenetics. For instance, the cell culture medium used in experiments contains fixed concentrations of nicotinamide which may not represent normal physiology and complicate understanding the relevant effects from supplementation with specific vitamin B3 metabolites. Nicotinamide-rich or -deficient media may alter flux through vitamin B3 metabolic pathways compared to traditional medium or medium containing nicotinic acid or NR. Also, the time at which measurements are taken will play a role in the observed effects, as metabolic and epigenetic effects vary in their time to manifest or may turnover faster than measurements are obtained.

Cell type is also an important determinant of the observed effects since the expression of and flux through enzymes participating in the vitamin B3 metabolome differ, as evidenced by the vastly different rates of NAD^+^ turnover. Careful consideration is required when selecting the model for study as the overall metabolic and respiratory differences between cell types is dramatic and will affect readout. Expression levels of the key enzymes participating in vitamin B3 metabolism should be examined and cell types selected based on the specific requirements of the experiment.

Despite these and other challenges, nonetheless, elucidating the intricate networks of vitamin B3 metabolism and epigenetics is essential for improving health care and offers potential opportunities for developing new diagnostic biomarkers and therapeutic tools for disease diagnosis and therapy. In particular, NNMT in different cancer subtypes appears to be a promising avenue as studies indicate a measurable “methyl-sink” effect relating to this enzyme that correlates with disease progression or aggressiveness. Measurements of SAM/SAH ratio or N-Me-NAM concentrations may inform on the metabolic status of specific cancer cells and indicate potential metabolic vulnerabilities that can be pharmacologically exploited. Also, the identification of nicotinic acid as a suppressor of tumor progression through the novel PTM lysine nicotinylation provides an entirely new biomarker that can be assessed. Nicotinic acid supplementation as part of the treatment or management of liver cancer may yield beneficial results and the discovery of this mechanism may lead to therapeutics that can elicit similar tumor suppressing effects.

## Author contributions

N. D. S.: conceptualization, writing, editing, visualization. A. G.: limited writing and editing. Y. G. Z.: conceptualization, review, editing, supervision, funding acquisition.

## Conflicts of interest

No conflicts of interest to declare.

## Data Availability

No new data were created, and all data analyzed in this review are available within the referenced literature.
